# A Pseudo‐*Mytilus Edulis* Foot Protein‐Based Hydrogel Adhesive with Osteo‐Vascular‐Immune Coupling Effects for Osteoporotic Bone‐Implant Integration

**DOI:** 10.1002/adma.202511840

**Published:** 2025-11-05

**Authors:** Wentao Wang, Zhenyu Li, Siming Zhang, Yue Ma, Lei Yu, Qidong Zhang, Guoqing Pan, Dechun Geng, Chen Zhu, Jiaxiang Bai

**Affiliations:** ^1^ Department of Orthopedics Centre for Leading Medicine and Advanced Technologies of IHM The First Affiliated Hospital of USTC Division of Life Sciences and Medicine University of Science and Technology of China Hefei Anhui 230022 P. R. China; ^2^ Department of Orthopedics Peking University First Hospital Beijing 100034 P. R. China; ^3^ School of Chemistry and Chemical Engineering Jiangsu University 301 Xuefu Rd Zhenjiang Jiangsu 212013 P. R. China; ^4^ Department of Orthopedics Qilu Hospital of Shangdong University Shandong University Jinan Shandong 250100 P. R. China; ^5^ Department of Orthopedics China‐Japan Friendship Hospital Beijing 100029 P. R. China; ^6^ Department of Orthopedics The First Affiliated Hospital of Soochow University 188 Shizi Street Suzhou Jiangsu 215006 P. R. China

**Keywords:** dual interface adhesive, immunomodulation, macrophage polarization, osteoporosis, pH‐responsive

## Abstract

The reduced initial stability of orthopedic implants in osteoporotic bone matrices, coupled with excessive M1 macrophage polarization at bone‐implant interfaces, disrupt bone–immune homeostasis and vascularization, ultimately leading to implant loosening or failure. Inspired by the marine mussel *Mytilus edulis* foot protein (Mefp), a pH‐responsive multifunctional bone glue (YDC‐Gel‐Zn) with broad‐spectrum adhesion capabilities is developed for osteoporotic bone‐implant integration. This pseudo‐Mefp bioglue enables dual‐interface adhesion via catechol‐rich sequences that mediate stable metal‐phenolic coordination with metallic implants and hydrogen‐bonded/Michael addition‐driven interactions with the bone matrix, thereby improving initial implant fixation. Under osteoporotic inflammatory microenvironments, sequential dissociation of borate ester bonds and metal‒phenolic coordination facilitates the controlled release of Zn^2^⁺ and proangiogenic/osteogenic peptides (YDC). The released Zn^2^⁺ remodels glutathione metabolism through glutathione S‐transferase (GST)‐mediated regulation of glutathione (GSH) levels, inhibits JAK1/STAT1/NLRP3 inflammasome activation, and suppresses the release of proinflammatory cytokines from senescent M1 macrophages, recalibrating the osteo‐vascular‐immune microenvironment. Due to its positive effects on bone regeneration and angiogenesis, the bioinspired bone bioglue demonstrated a 194% increase in fixation strength in osteoporotic rat models, achieving 93% healthy bone‐implant stability. Overall, this study provides a clinically translatable strategy for stable implantation under osteoporotic conditions through synergistic mechanical adaptation, bioactivity regulation, and smart environmental responsiveness.

## Graphical abstract

Inspired by mussel proteins, YDC‐Gel‐Zn is a smart bone glue designed for osteoporotic implants. It adheres strongly to both metal implants and bone for initial fixation. In osteoporosis's inflammatory environment, it sequentially releases zinc ions and therapeutic peptides: the zinc modulates immune cells to reduce inflammation, while the peptides stimulate bone regeneration and blood vessel formation. This dual‐action approach enhances implant stability in osteoporotic bone, achieving near‐healthy bone performance.

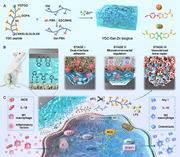

## 1 Introduction

Osteoporosis, a systemic skeletal disorder affecting over 200 million people worldwide, manifests as bone mass deterioration and microarchitectural disruption, substantially increasing the risk of fracture.^[^ ^1^ ^]^ Titanium and its alloys have emerged as the most extensively utilized implant materials in clinical practice owing to their chemical stability, superior biocompatibility, high mechanical strength, and lightweight properties. However, the inherent bioinertness of titanium‐based materials not only precludes their inherent osteogenic and angiogenic capacities but also renders traditional surface modification techniques complex and demanding in terms of processing conditions.^[^ ^2^ ^]^ In patients with osteoporosis, profound bone loss and concomitant disruption of the peri‐implant immune microenvironment not only impede the intimate integration of modified materials with the bone matrix, thereby compromising their functional efficacy, but also exacerbate the inhibition of osteogenesis and angiogenesis by inducing a localized inflammatory milieu.^[^ ^3^, ^4^ ^]^

The following post‐implantation inflammatory paradox presents a critical challenge: acute inflammation at the bone‐implant interface, driven by M1 macrophage polarization, is essential for initiating angiogenesis and stem cell recruitment; however, persistent activation exacerbates immune dysregulation and accelerates macrophage senescence, thereby suppressing M2 transition.^[^ ^5^, ^6^ ^]^ In osteoporotic bones, which are characterized by diminished bone mineral density and compromised vascularity‐this dysregulated phase impedes the reparative triad of inflammation‐resolution, angiogenesis, and osteogenic remodeling, but mechanical instability from bone loss increases the risk of early loosening. Osteoporosis is characterized by bone loss and persistent inflammation. Macrophage‐mediated immune dysregulation creates a hostile microenvironment for implant integration. Furthermore, angiogenesis is impaired because of reduced VEGF signaling and endothelial dysfunction, leading to poor bone formation.^[^ ^7^, ^8^ ^]^ Although bone‐cement augmentation provides temporary fixation, its bioinert nature fails to address core pathological cascades, such as the absence of osteoinductivity, chronic inflammation‐induced non‐degradability, and inability to modulate immune‐vascular coupling.^[^ ^9^, ^10^ ^]^ Consequently, the development of phase‐adaptive biomaterials that synchronize mechanical stabilization with spatiotemporal immunometabolic reprogramming is imperative to reverse osteoporosis‐imposed integration failure.

In this study, inspired by Mefps, we engineered a mussel‐derived osteogenic‐angiogenic fusion peptide (YDC) through rational biomimicry. Mefps achieve robust underwater adhesion via 3,4‐dihydroxyphenylalanine (DOPA), whose catechol moieties enable covalent cross‐linking, hydrogen bonding, and metal‐phenolic coordination across diverse interfaces (e.g., bone tissue and titanium).^[^ ^11^, ^12^ ^]^ Through biomimetic design, we conjugated YGF (YGFGG) and CK peptides (CKKSLSLSLSLKK) via the strongly adhesive DOPA group to create a YDC mussel‐inspired peptide. The YGF peptide represents the minimal amino acid sequence that retains full osteogenic growth peptide (OGP)‐like activity, preserving most of the biological activity of natural OGP while demonstrating enhanced stability, whereas the CK peptide is a proangiogenic peptide functionally analogous to the YGF peptide. Using gelatin (Gel), a biocompatible natural polymer, as the backbone structure, phenylboronic acid (PBA) was subsequently grafted onto side chains.^[^ ^13^ ^]^ By leveraging the dynamic reversible boronate ester bonds formed between the PBA and catechol groups, we integrated the YDC peptide with a gel backbone to construct a cross‐linked network system. To further increase the mechanical strength of the bioglue, metal ions were introduced to establish metal‒phenolic coordination cross‐linking between YDC peptides, forming an engineering‐inspired trus‐like composite system. This design not only reinforced the multidimensional stability of the bioglue but also incorporated the immunomodulatory properties of metal ions (Zn^2^⁺), ultimately yielding a self‐healing bioglue system designated as YDC‐Gel‐Zn.

**Figure 1:**
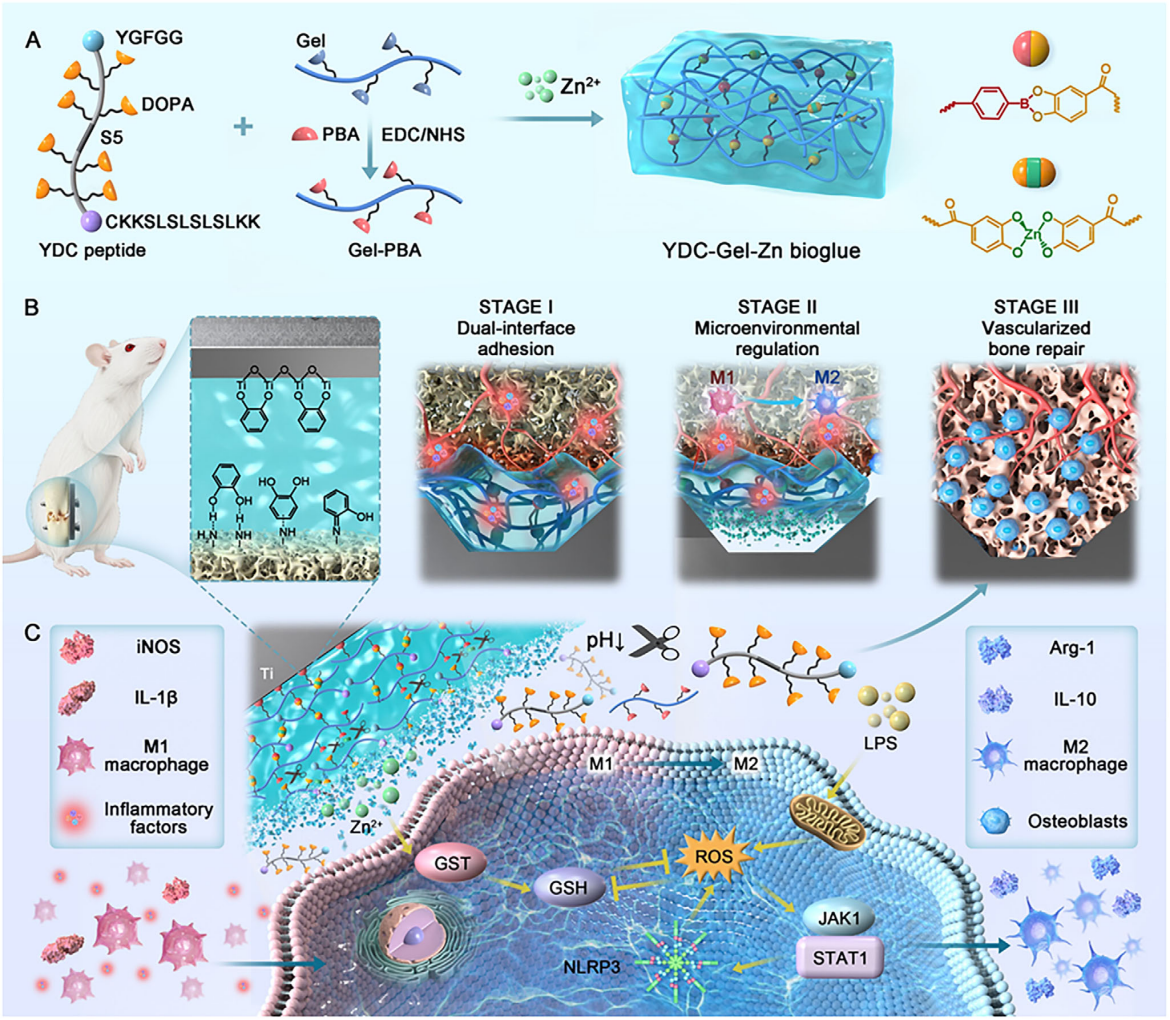
Synthesis of YDC‐Gel‐Zn and its dual‐interface adhesion and sequential regulation in promoting bone‐implant osseointegration. A) Schematic illustration of the structures of YDC and Gel‐PBA, as well as the YDC‐Gel‐Zn bioglue formed via boronic ester bonds and metal‐phenolic coordination cross‐linking. B) Dual‐interface adhesion of the bio‐glue and the three stages of implant‐bone surface integration during intrafemoral implantation in osteoporotic rats. C) Immunomodulatory biological mechanism of the pH‐responsive bioglue via the GST/ROS/JAK1/STAT1/NLRP3 signaling pathway.

Synthetic bioglue hydrogels have multiple functions and can provide mechanical stabilization of intraosseous implants during the initial fixation phase while exhibiting pH‐responsive dissolution characteristics during the inflammatory phase, thereby enabling spatiotemporally controlled sequential release of the YDC peptide and Zn^2^⁺ ions (**Figure** **1**). Mechanistically, the released Zn^2^⁺ modulates glutathione metabolism via GST‐mediated pathways, effectively scavenging reactive oxygen species (ROS) and suppressing the activation of JAk1/STAT1/NLRP3 inflammasomes. This synergistic immunomodulatory effect attenuates macrophage senescence and M1 polarization, thereby rectifying the immune imbalance at bone–implant interface and establishing a pro‐regenerative microenvironment that potentiates the osteogenic and angiogenic effects of the YDC peptide. Overall, the YDC‐Zn hydrogel system exhibited superior osseointegration‐promoting efficacy under osteoporotic conditions through a tripartite mechanism, which involved biomechanical stabilization, immunomodulation, and coupled osteogenesis‐angiogenesis regulation.

## 2 Results

### 2.1 Synthesis and Characterization of YDC‐Gel‐Zn Bioglue

**Figure 2:**
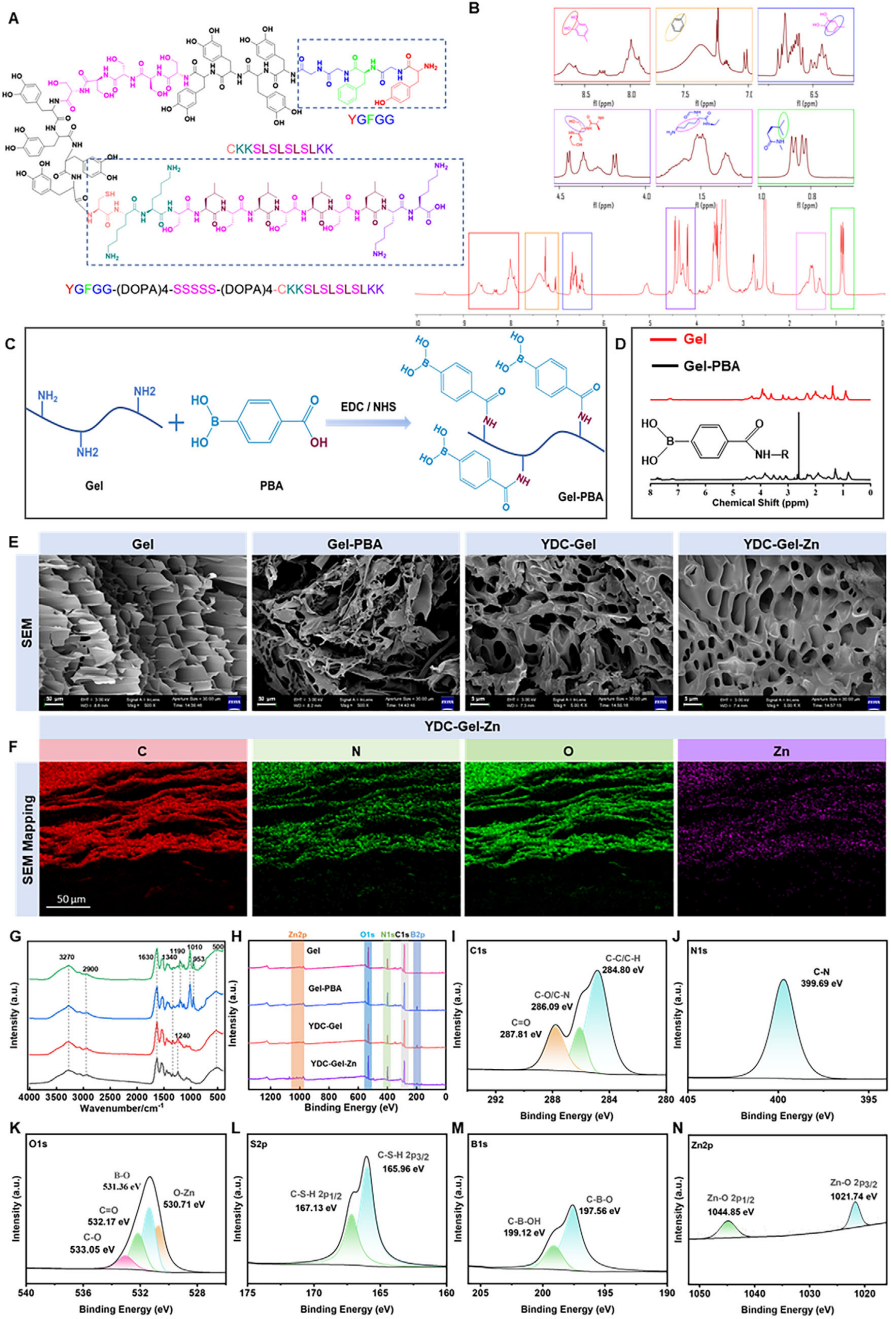
Characterization of the bioglue. A) Schematic illustration of the structure of YDC mussel‐derived peptide. B) ^1^H NMR spectrum of the YDC mussel‐derived peptide. C) Schematic illustration of the synthesis process for Gel‐PBA. D) ^1^H NMR spectrum of Gel‐PBA. E) SEM images of Gel, Gel‐PBA, YDC‐Gel, and YDC‐Gel‐Zn. F) Elemental mapping analysis of YDC‐Gel‐Zn. G) FTIR spectra of Gel, Gel‐PBA, YDC‐Gel, and YDC‐Gel‐Zn. H–N) XPS survey spectra of Gel, Gel‐PBA, YDC‐Gel, and YDC‐Gel‐Zn and detailed spectral analyses of YDC‐Gel‐Zn.

In this study, we initially focused on the molecular design of bioinspired active peptides, employing Fmoc‐solid‐phase synthesis to link the YG (YGFGG) and CK (CKKSLSLSLSLKK) peptides with DOPA and serine (S). Finally, we synthesized the YDC peptide [YGFGG‐(DOPA)_4_‐SSSSS‐(DOPA)_4_‐CKKSLSLSLSLKK] (**Figure** **2**A). In the YDC peptide, the YG peptide, as the C‐terminal pentapeptide sequence of the OGP, not only retains natural osteogenic activity but also reduces immunogenicity and synthetic difficulty.^[^ ^14^, ^15^ ^]^ The CK peptide exhibits excellent pro‐angiogenic capacity and is capable of promoting vascular regeneration by stimulating endothelial cell proliferation.^[^ ^16^ ^]^ The DOPA groups not only provide the catechol structure required for YDC grafting onto Gel‐PBA and dual‐interface adhesion, but also enable free radical scavenging (via the DOAP groups) to protect the peptide segments.^[^ ^17^ ^]^ Serine residues within the peptide segments serve as linkers to ensure the independent functionality of each component and impart stability and flexibility to the synthetic polypeptide. Additionally, polar hydroxyl groups of serine enhance the solubility of hydrophobic peptide segments.^[^ ^18^ ^]^ Subsequently, we comprehensively characterized and analyzed the synthesized YDC. The results of the nuclear magnetic hydrogen spectrum (1H NMR) analysis are shown in Figure 2B; the signals of the active protons in the structure located in the low‐field region on the left; a red box highlights the phenolic hydroxyl protons, while yellow and blue boxes correspond to benzene ring protons. The high‐field region represents the signal range of alkyl protons, with a purple box near 4.3 ppm indicating protons of alkyl, hydroxyl, and attached methylene groups. The peak near 1.6 ppm corresponds to the methylene proton signal of the alkyl chain, and the signal at 0.9 ppm represents the terminal methyl protons of the alkyl chain. The characteristic peaks in the nuclear magnetic hydrogen spectrum corresponded well with the structural formula of the YDC peptide.

We subsequently conducted electrospray ionization mass spectrometry (ESI‐MS) analysis on the YDC peptide (Figure S1A, Supporting Information), with the isotopic mass numbers [M+5H]^5+^ and [M+4H]^4+^ of the YDC peptide appearing at 758.30 Da and 947.74 Da, respectively, which correspond to the theoretical molecular weight of the YDC peptide of 3785.24 Da. Additionally, we performed high‐performance liquid chromatography (HPLC) analysis on the synthesized YDC peptide, and the results indicated that the purity of the synthesized peptide was >95% (Figure S1B, Supporting Information). In summary, we successfully synthesized a high‐purity YDC bio‐peptide.

We then utilized the Gel as a vehicle for the hydrogel bioglue and grafted PBA onto its side chains. The Gel, which is a significant component of the extracellular matrix, is not only cost‐effective and readily available but also has excellent biocompatibility and nonimmunogenicity.^[^ ^19^ ^]^ Moreover, the rich amino groups on the Gel side chains allow the grafting of PBA onto the Gel under mild conditions, resulting in the synthesis of Gel‐PBA (Figure 2C). 1H NMR results revealed a characteristic peak at 2.6 ppm for Gel‐PBA, indicating the successful grafting of PBA onto the Gel (Figure 2D). A quantitative nuclear magnetic resonance (qNMR) analysis demonstrated that the PBA content of Gel‐PBA was 20%, further confirming the successful and effective grafting of PBA (Figure S1C, Supporting Information). We then dissolved 60 mg of lyophilized Gel‐PBA in 300 µL of PBS, with or without Zn^2^⁺ ions. The dissolved Gel‐PBA/Gel‐PBA‐Zn appeared liquid‐like. Then, we thoroughly mixed the dissolved Gel‐PBA/Gel‐PBA‐Zn solution using a vortex oscillator at 2000 rpm. During vortex oscillation, we continuously added 100 µL of a 0.1 g µL^−1^ YDC peptide mixture. This rapidly transformed the vortexing mixture into a translucent hydrogel. Inversion experiments demonstrated that YDC rapidly formed a stable hydrogel in the Gel‐PBA/Gel‐PBA‐Zn solution, which remained at the bottom of the centrifuge tube without flowability (Figure S1D, Supporting Information).

Theoretically, PBA grafted onto the Gel side chains can rapidly cross‐link with the catechols in the YDC peptide under mild conditions to form boronate esters, whereas the catechol structures in YDC can tightly bind to metal Zn ions through metal‒phenolic coordination,^[^ ^20^ ^]^ ultimately forming a YDC‒Gel‒Zn hydrogel network (Figure S2A, Supporting Information). To theoretically investigate whether YDC and Zn^2^⁺ cross‐link with Gel‐PBA through chemical bonds rather than mere physical binding, we first employed scanning electron microscope (SEM) to analyze the microporous structures of Gel, Gel‐PBA, YDC‐Gel, and YDC‐Gel‐Zn after freeze‐drying (Figure 2E). The pure‐Gel group exhibited a uniform square pore structure. After grafting with PBA, the structure of Gel‐PBA changed significantly and exhibited an unstable reticular structure composed of disordered porous flakes. YDC‐Gel, due to the addition of mussel‐derived peptides capable of forming borate ester bonds, displayed a remarkably regular and dense porous reticular structure. In addition, there were no significant differences between the structures of the YDC‐Gel‐Zn and YDC‐Gel groups. EDS results of the YDC‐Gel‐Zn group (Figure 2F) indicated that Zn was successfully and uniformly grafted onto the bioglue. These results demonstrate that mussel‐derived peptide YDC and Zn^2^⁺ cross‐link with Gel‐PBA through chemical bonding rather than simple physical binding.

We then conducted Fourier transform infrared (FTIR) spectroscopy and X‐ray photoelectron spectroscopy (XPS) analyses to validate the formation mechanism of the YDC‐Gel‐Zn bioglue. FTIR results (Figure 2G) indicated that all four groups of hydrogels presented characteristic peaks at 3270 cm^^−1^ and ≈2900 cm^^−1^, corresponding to corresponding to the stretching vibrations of O─H and C─H, respectively, which are typical of organic compounds. The peak at 1630 cm^^−1^ corresponded to the stretching vibration of C═O, a functional group typical of Gel. In the Gel and Gel‐PBA groups, the peak at 1240 cm^^−1^ was primarily associated with the presence of C─O. However, in the YDC‐Gel and YDC‐Gel‐Zn groups, the characteristic peak shifted to 1190 cm^^−1^ due to coordination with the peptide, and the peaks at 1010 and 953 cm^^−1^ in the YDC‐Gel and YDC‐Gel‐Zn groups were associated with the out‐of‐plane bending vibrations of C‐H in phenyl rings. The abundance of phenyl rings in the peptide structure explains the significant increase in characteristic peaks at these locations for the YDC‐Gel and YDC‐Gel‐Zn groups. Compared with the Gel and Gel‐PBA groups, the YDC‐Gel and YDC‐Gel‐Zn groups did not show significant peak shifts; however, the YDC‐Gel‐Zn group exhibited increased peak intensity at approximately 500 cm^^−1^, primarily associated with metal‒oxygen bonds (M─O), indicating the formation of Zn─O bonds due to the addition of Zn, suggesting the coordination of Zn with phenolic hydroxyl groups.

XPS results (Figure 2H) further confirmed that, compared with the Gel group, the Gel‐PBA group exhibited the characteristic peak of B2p at 197 eV, and similar to the YDC‐Gel group, the YDC‐Gel‐Zn group also presented Zn2p peaks at 1044 and 1021 eV. Subsequent analyses of the peak values for different elements in the YDC‐Gel‐Zn group (Figure 2I–N) revealed that in the C1s XPS spectrum, three distinct peaks were clearly observed at 284.80, 286.09, and 287.81 eV, corresponding to C─C/C─H, C─O/C─N, and C═O bonds, respectively. These peaks, which are characteristic of Gel, PBA, and peptides, not only validate the coexistence of these components but also imply their intimate association within the material structure. The C─O bond probably originated from the cleavage of OH bonds in PBA and their subsequent binding to carbon atoms on the peptide to form C─O bonds or from the cleavage of phenolic OH bonds on the peptide chain, with the departure of hydrogen atoms and the remaining oxygen atoms coordinating with Zn^2^⁺ ions to form C─O bonds. The C─N bond was primarily attributed to the presence of nitrogen atoms in Gel and peptides. Most of the C═O bonds originate from the peptide, further confirming their significant role in material structure. In the O1s spectrum, four distinct peaks were identified at 530.71, 531.36, 532.17, and 533.05 eV, corresponding to O─Zn, B─O, C═O, and C─O bonds, respectively. The presence of Zn─O validated the successful coordination of Zn^2^⁺ ions. Moreover, in the N1s spectrum, the existence of C─N bonds was clearly observed, further confirming the contribution of Gel and peptides to the material structure. In the B1s spectrum, two significant peaks were attributed to the presence of C─B─O and C─B─OH bonds. The appearance of C─B─O not only provides strong evidence of the presence of borate esters but also reveals the unique role of boron atoms within the material structure. In addition, in the S2p spectrum, two distinct peaks were observed, corresponding to C─S─H bonds in the peptide component. Finally, in the Zn2p spectrum, distinct peaks were observed, corresponding to the binding energies of the 2p3/2 and 2p1/2 orbitals of Zn─O bonds formed by the coordination of Zn^2^⁺ ions with phenols. This finding is consistent with the results from the O1s spectrum, indicating the coordination relationship between Zn^2^⁺ ions and phenolic compounds.

### 2.2 Rheological and Self‐Healing Properties of the Bioglue

**Figure 3:**
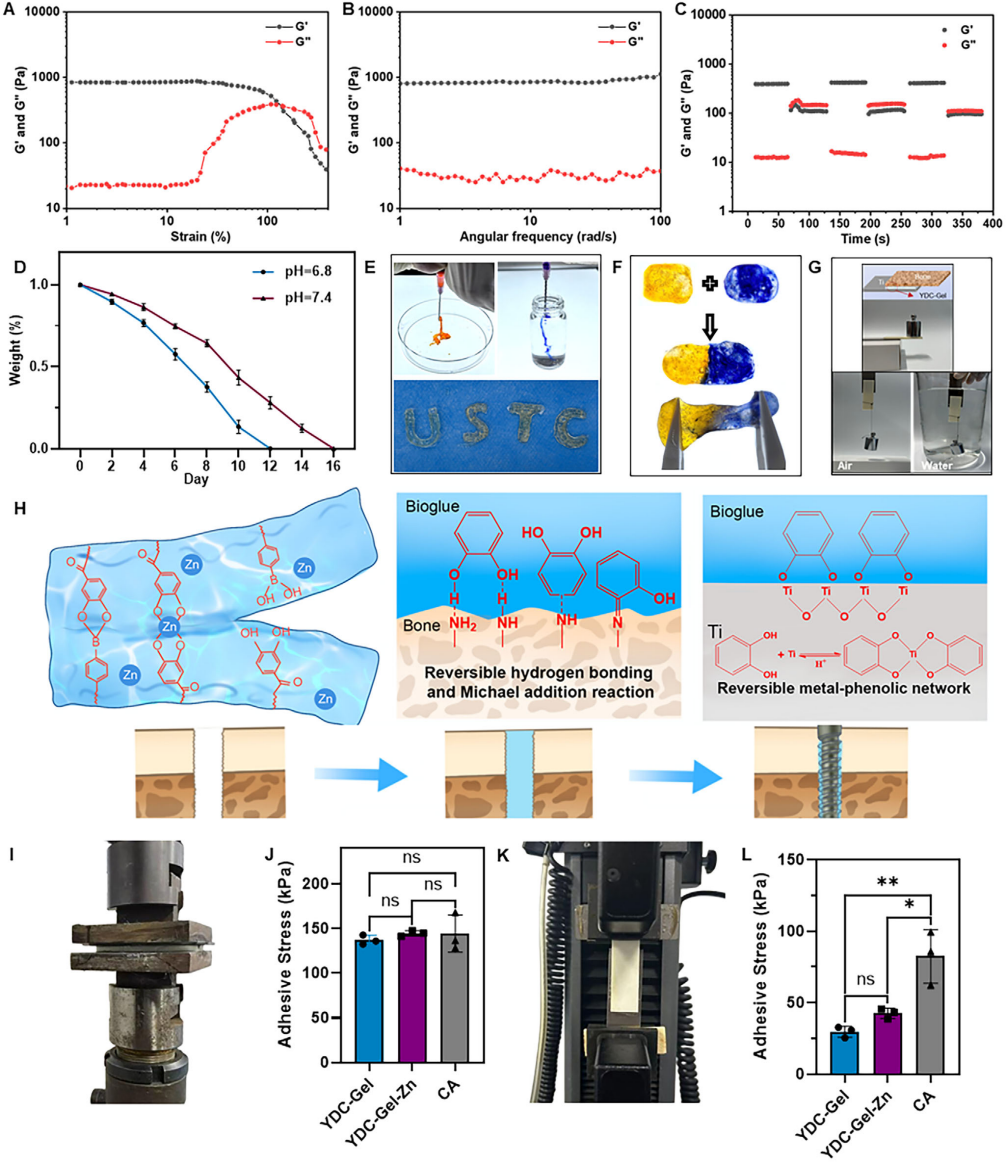
Dual interface adhesive and hemostatic properties of the bioglue. A–C) Frequency sweep, strain sweep, and time‐dependent rheological profiles of YDC‐Gel‐Zn, showing the storage modulus (G′) and loss modulus (G″). D) Dissolution kinetics of YDC‐Gel‐Zn at different pH values (*n* = 3). E) Self‐healing ability of YDC‐Gel‐Zn bioglue. F) Injectable behavior and moldability of YDC‐Gel‐Zn bioglue. G) Schematic illustration of YDC‐Gel‐Zn application and its dual‐interface adhesion mechanism. H) Double interface adhesion ability of the bioglue in dry and wet environments. I–J) Tensile testing of YDC‐Gel, YDC‐Gel‐Zn and CA at the bone plate‐titanium sheet interface. K–L) Evaluation of the shear strength of YDC‐Gel, YDC‐Gel‐Zn and CA at the bone plate‒titanium sheet interface. The data are presented as the means ± SDs in the analysis figures, with *p < 0.05 and **p < 0.01 indicating statistical significance.

The structural stability and self‐healing capability of bioglue are fundamental for achieving double‐interface adhesion.^[^ ^21^ ^]^ Hydrogels with poor structural stability and inability to self‐heal are prone to damage under significant stress, which not only fails to achieve the desired therapeutic effect but can also easily lead to local inflammation, exacerbating the microenvironmental disorder at the bone–implant interface.^[^ ^22^ ^]^ We assessed the rheological properties of the bioglue to evaluate the self‐healing ability and structural strength of the synthesized bioglue by examining the relationships between the storage modulus (G′) and loss modulus (G′′) of YDC‐Gel‐Zn and the strain and angular frequency. As shown in **Figure** **3**A, within the low strain range, the storage modulus (G′) and loss modulus (G″) of YDC‐Gel‐Zn remained relatively constant, with G′ consistently higher than G″, indicating that the material exhibited predominantly elastic behavior, which enabled it to resist certain mechanical stresses and maintain its Gel state. When the strain reached the critical strain region (23%), G′ decreased rapidly, while G″ increased significantly. Finally, at the critical value (140%), G″ exceeded G′, indicating that the material underwent structural failure under large strain, transitioning from an elastic‐dominated gel to a viscous‐dominated sol. In a frequency sweep analysis (Figure 3B), both G′ and G″ did not show any significant amplitude change, indicating that the material possessed a stable elastic network and good resistance to frequency‐induced structural rearrangements. In a continuous step strain analysis (Figure 3C), G′ and G″ of YDC‐Gel‐Zn remained stable within the test time (0–400 s), with no obvious upward or downward trends, demonstrating that the material's structure was stable over time, with no spontaneous cross–linking or destruction. Moreover, G′ remained higher than G″ throughout the testing duration, maintaining the elastic gel state. These results indicate that YDC‐Gel‐Zn not only has a stable structure but also possesses good self‐healing ability. This forms the basis for injectability and, in clinical practice, the bioglue—when injected into the nail canal in advance, to transition from a solid gel to a liquid sol when faced with the significant stress of screw insertion. It closely conforms to the bone defect gaps and the surface of the implant, avoids strong stress, and then transforms back into a solid gel, achieving rational redistribution of the bioglue. As depicted in Figure 3E,F, the injectable YDC‐Gel‐Zn bioglue can be injected into molds of various shapes to control the shape of the gel. By relying on the reversible cross‐linking of boronate ester bonds, cutting the self‐assembled bioglue in half and then reconnecting it for 5 min can achieve self‐healing. In summary, mussel‐derived bioglue has outstanding structural strength and self‐healing ability, meeting the clinical demands for self‐healing and injectability.

Next, the bioglue degradation rate was assessed in vitro. Initially, PBS (pH 7.4) was used to simulate the normal physiological microenvironment of the human body. Under weakly alkaline conditions, the bioglue exhibited sustained and stable degradation over 16 days. However, the cellular microenvironment was weakly acidic.^[^ ^23^, ^24^ ^]^ Therefore, we also evaluated the dissolution rate of the bioglue in weakly acidic PBS at pH 6.8. Figure 3D showed that the bioglue completely dissolved within 12 days under weakly acidic conditions, which can be attributed to the instability of borate ester bonds and metal‒phenolic coordination in acidic environments.^[^ ^25^, ^26^ ^]^ Under acidic conditions, hydrogen ions readily react with oxygen atoms in the borate ester, leading to polarized cleavage of the boron‐oxygen bonds. Additionally, the hydroxyl groups (─OH) of phenolic ligands are prone to protonation (forming ─OH_2_⁺) under acidic conditions, which weakens the coordination bonds between the metal and the ligands, potentially causing dissociation of the complex.^[^ ^27^, ^28^ ^]^ Nevertheless, due to the relatively low concentration of hydrogen ions in weakly acidic environments, combined with the chelation effect introduced by ortho‐hydroxyl groups in the borate ester and the multiple coordination sites provided by polyphenolic ligands, the stability of the bioglue is enhanced. This allows the bioglue to achieve pH‐responsive sustained release in inflammatory environments, enabling it to function more precisely during the inflammatory phase of bone repair.

During the dissolution of the bioglue, we detected the release dose of the YDC peptide and zinc ions (Figure S3A–B, Supporting Information). We found that YDC and zinc ions gradually released with the dissolution of the bioglue, and the release rate of zinc ions was faster than that of the YDC peptide in neutral environment, however, in acidic environment, the release rates of both were similar. This may be related to the stronger reversible binding ability of borate ester bond under mild conditions, which also realized the sequential controlled release of zinc ion and YDC peptide.^[^ ^29^, ^30^ ^]^

### 2.3 Adhesive Performance of the Bioglue

Owing to the strong adhesion of the DOPA groups in the mussel‐derived peptides, the synthesized YDC‐Gel‐Zn bioglue exhibited good ductility while maintaining excellent adhesion (Figure S4A, Supporting Information). It firmly adhered not only to the surface of rubber gloves, but also to the surfaces of fresh liver, heart, and bone tissues (Figure S4B, Supporting Information). Moreover, the bioglue remained firmly attached to the glove surface without detachment during bending and torsion (Figure S4C, Supporting Information). The adhesion of the bioglue at the dual interfaces of the Ti‐based material surface and bone tissue is necessary for achieving initial stability of the implant. Theoretically, catechol groups in DOPA can form coordination bonds with Ti ions in Ti‐based material, enhancing the adhesion between the bioglue and the Ti‐based material. Additionally, the catechol groups can not only form hydrogen bonds with the bone tissue surface and form chemical bonds with amine or thiol groups through Michael addition and Schiff base reactions, strengthening the adhesion between DOPA and bone tissue (Figure 3H).

The double‐interface adhesion ability of the biological glue is essential for stable implantation.^[^ ^31^ ^]^ To further verify the adhesion ability of the bioglue between bone tissue and the Ti‐based material, we first evenly coated the bioglue on the surface of untreated bovine bone slices and then pressed it onto the surface of a Ti plate. After standing at 25° for 3 min, the bone plate and Ti sheet adhered by the bioglue could withstand a 200 g weight in the vertical direction and horizontal pulling force. Moreover, good adhesion ability was maintained in a humid environment (Figure 3G). We subsequently applied the bioglue between a bone slice and Ti plate and further tested the adhesion ability of the bioglue via vertical tensile (ASTM F2258‐05) (Figure 3I–J) and lap shear tensile tests (ASTM F2255‐05) (Figure 3K,L). Meanwhile, for comparison, we chose commercially available medical glue as the positive control. Its primary component is liquid cyanoacrylate (CA), which is widely used in clinical practice for skin wound adhesion. The vertical and shear tensile strengths were both relatively high in the YDC‐Gel‐Zn group, reaching 137.02 ± 4.24 kPa (Figure S5A–B, Supporting Information)and 29.68 ± 3.08 kPa, respectively (Figure S5D,E, Supporting Information), and the endpoints of both tests were the fracture of the hydrogel (Figure S6G,H, Supporting Information). The tensile and shear strengths of the CA group were also high. Its tensile strength was 144.22 ± 17.09 kPa (Figure S5C, Supporting Information), similar to that of the bioglue group, while its shear strength was 82.38 ± 15.37 kPa (Figure S5F, Supporting Information), higher than that of the bioglue group. However, their curves differed from those of the bioglue. This is because the bonding method primarily involved a rapid anionic polymerization reaction that instantly transformed liquid monomers into solid polymers through mechanical interlocking, thereby adhering objects. Therefore, it suddenly fractured after withstanding the high‐strength stress. However, owing to the chemical bonding characteristic of the bioglue, the reversible chemical cross‐linking of YDC‐Gel and YDC‐Gel‐Zn enabled it to effectively disperse stress when subjected to external forces, resulting in ductile fracture. Therefore, the endpoint of both bioglue tests were hydrogel fracture. These results indicate that the bioglue has excellent dual‐interface adhesion capability.

In clinical practice, when performing orthopedic internal screw fixation, it is necessary to predill holes in the bone, which inevitably damages the blood vessels in the periosteum and bone marrow cavity, resulting in bleeding. This is also the main cause of hematoma formation at the fracture site.^[^ ^32^ ^]^ However, a hematoma in the screw tract not only increases the risk of infection but also attracts inflammatory cells such as macrophages to the hematoma site. Although the hematoma gets absorbed, many inflammatory mediators are released, aggravating disorders in the local immune microenvironment.^[^ ^33^ ^]^ Therefore, biomaterials with good hemostatic abilities are crucial for promoting osteointegration. In this study, we investigated the hemostatic performance of YDC‐Gel‐Zn bioglue.

As shown in the hemostasis schematic diagram of the bioglue (Figure S6A, Supporting Information), the bioglue based on mussel‐derived peptides could quickly adhere to the damaged site to form a physical barrier for hemostasis and accelerate blood coagulation by promoting the activation of blood coagulation factors and platelets.^[^ ^34^, ^35^ ^]^ In rat liver hemorrhage models (Figure S6B–C, Supporting Information), YDC‐Gel and YDC‐Gel‐Zn showed significantly reduced blood loss versus commercial hemostatic sponges at 30 and 60s, with no intergroup difference. In vitro whole‐blood coagulation assays (Figure S6D,E, Supporting Information) revealed superior clotting capability of both bioglues over controls at 10 and 30 min. Notably, YDC‐Gel‐Zn exhibited enhanced coagulation versus YDC‐Gel, forming stable clots by 30 min. The observed in vitro–in vivo discrepancy may arise from Zn^2^⁺‐driven fibrin clot acceleration via platelet activation—a process requiring longer timescales than captured in acute hemorrhage models.^[^ ^36^ ^]^ In general, these results indicate that YDC‐Gel‐Zn bioglue also has excellent hemostatic ability, which meets the hemostatic requirements for orthopedic internal implantation and has the potential to be transformed into a hemostatic wound dressing.

### 2.4 Biocompatibility Properties of the Bioglue

Prior to conducting in vitro and in vivo experiments to investigate the osteogenic, angiogenic and immunomodulatory effects of YDC‐Gel‐Zn, we first evaluated the biocompatibility of the YDC‐Gel‐Zn bioglue to assess its safety for clinical applications. The bioglue (100 µL) was completely dissolved in 1, 2, and 4 mL of a culture medium to prepare extraction solutions with YDC concentrations of 1.32, 0.66, and 0.33 µM, respectively. After bone marrow‐derived macrophages (BMMs) (Figure S7A, Supporting Information) and bone marrow mesenchymal stem cells (BMSCs) (Figure S7B, Supporting Information) were co‐cultured with these extraction solutions for 1, 3, and 5 days, live/dead staining demonstrated that none of the three concentrations exhibited significant cytotoxicity (Figure S8A, Supporting Information). The results of CCK‐8 assay further confirmed that these extraction solutions did not affect cell proliferation, indicating their excellent biocompatibility (Figure S8B,C, Supporting Information). Based on these results, we selected the 1.32 µM bioglue extraction solution for subsequent cellular experiments. In previous studies, we evaluated the hemostatic ability of the bioglue. Therefore, we also tested the hemocompatibility of the bioglue to determine whether damaged red blood cells. Deionized water and PBS were used as positive and negative controls, respectively. The experimental results (Figure S8D,E, Supporting Information) revealed that the supernatants of the YDC‐Gel and YDC‐Gel‐Zn groups were similar to those of the PBS‐negative control group, suggesting that the bioglue groups showed no significant hemolysis of red blood cells. A absorbance of the supernatant from each group was measured at 450 nm. The absorbance values of the PBS, YDC‐Gel, and YDC‐Gel‐Zn groups were all ≈0.05, whereas that of the positive control group at 450 nm was greater than 1.0. These results suggest that the bioglue exhibits good biocompatibility and hemocompatibility.

### 2.5 Immune Regulation Property of the Bioglue

**Figure 4:**
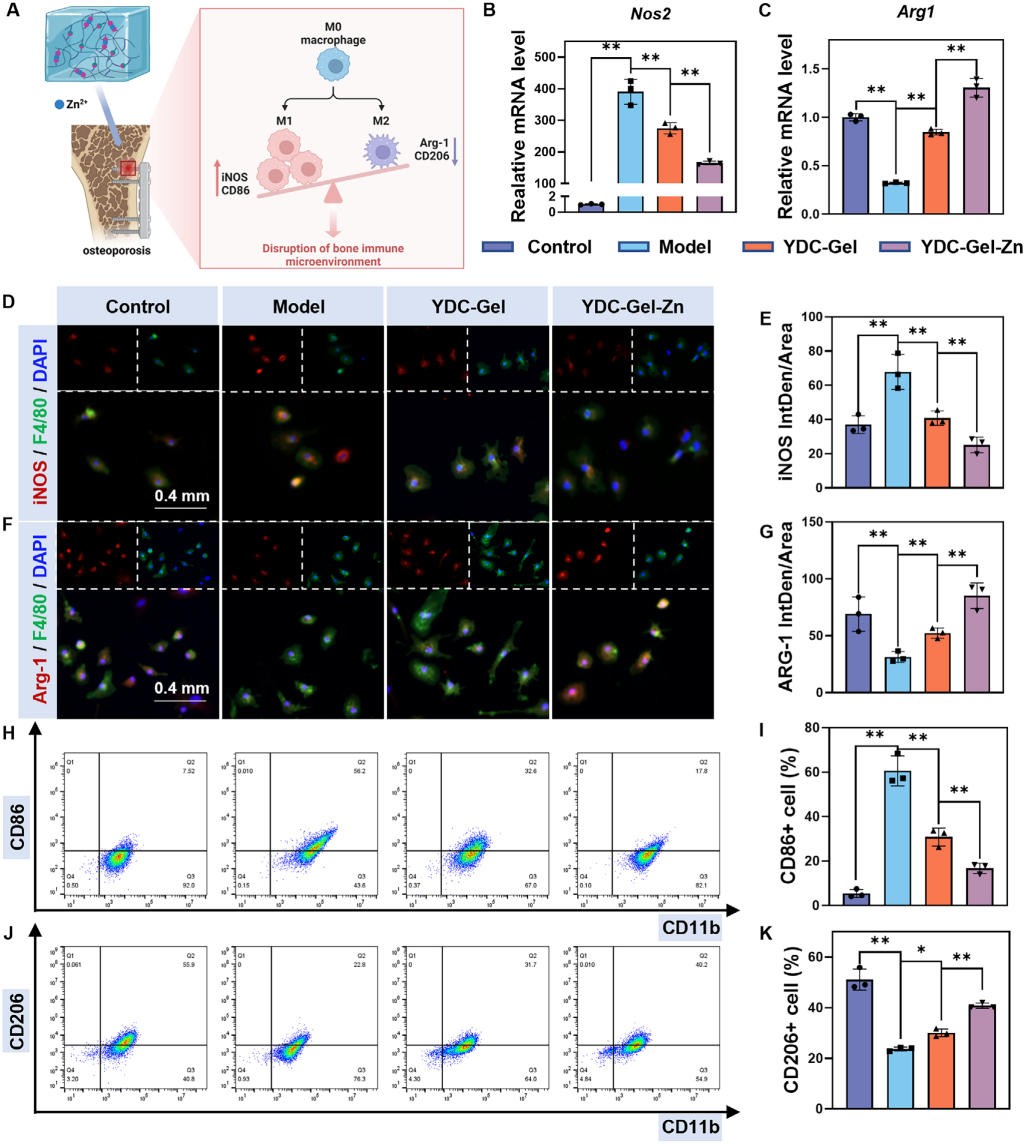
In vitro regulation of macrophage polarization by bioglue. A) Schematic illustration of bioglue modulation of the local osteoimmune microenvironment via macrophage polarization regulation at the bone–implant interface (created with BioRender.com). B–C) RT‒qPCR analysis of Nos2, and Arg1 expression levels in BMMs across different groups, along with quantitative results (*n* = 3). D–G) Immunofluorescence staining of BMMs to assess macrophage polarization (macrophage marker F4/80: green; M1 marker iNOS: red; M2 marker CD206: red; nuclei: blue) and quantitative analysis of immunofluorescence staining (*n* = 3). H,K) Flow cytometry analysis of macrophage polarization in BMMs (macrophage marker CD11b, M1 marker CD86, and M2 marker CD206) and corresponding quantification (*n* = 3). The data are presented as the means ± SDs in the analysis figures, with *p < 0.05 and **p < 0.01 indicating statistical significance.

Clinically, the hematomas and bone debris generated during the implantation of internal fixation materials in patients with fractures can induce aseptic inflammation at the bone‐implant interface, recruiting macrophages to engulf these hematomas and bone debris.^[^ ^37^ ^]^ This process is accompanied by a massive release of inflammatory cytokines, which further exacerbates chronic inflammation in osteoporotic patients and severely disrupts bone integration at the bone–implant interface in osteoporotic patients.^[^ ^38^ ^]^ The YDC‐Gel‐Zn bioglue gradually releases Zn^2^⁺ ions with immunomodulatory properties in a low‐pH environment during local inflammation,^[^ ^39^ ^]^ which can modulate macrophage polarization to alleviate the disordered local immune microenvironment (**Figure** **4**A).

We investigated the immunomodulatory capacity of the bioglue using BMMs in vitro (Figure S9A, Supporting Information). As shown in Figure 4B,C and Figure S9B,C (Supporting Information), PCR results indicated that the macrophages in the Model group exhibited significant M1 polarization (Nos2 and IL‐1β) under the intervention of lipopolysaccharide (LPS), whereas the expression of phenotypic markers of M2 macrophages (Arg‐1 and IL‐10) was significantly reduced. In contrast, the M1 polarization of macrophages was inhibited in the YDC‐Gel group, probably because of the DOPA groups in the peptide structure of YDC. The DOPA groups not only confer adhesive properties to YDC peptides but also, as polyphenolic structures, exert antioxidant stress effects and play a role in immune regulation. Compared with the YDC‐Gel group, the YDC‐Gel‐Zn group presented significantly lower expression of M1 macrophage marker genes and markedly higher expression of M2 macrophage marker genes. We subsequently performed immunofluorescence co‐staining of the M1/M2 macrophage‐related proteins and the macrophage surface marker F4/80 in each group, and the results were consistent with those of a western blot analysis (Figure 4D–G). Previous studies have demonstrated that M2 macrophages can also secrete osteogenic‐related cytokines to regulate of the bone microenvironment.^[^ ^40^, ^41^ ^]^ A qPCR analysis of the expression levels of osteogenic‐related genes, revealed that, similar to the M2 phenotype, the expression levels of TGF‐β and PDGF were significantly decreased in the Model group but remarkably increased in the YDC‐Gel‐Zn group (Figure S9D,E, Supporting Information). These findings indicate that bio‐glue not only participates in immune regulation and exerts immunomodulatory and osteogenic effects.

We also conducted flow a cytometry analysis of the surface markers CD86 (M1 macrophage marker), CD206 (M2 macrophage marker) and CD11b (macrophage surface marker) to assess the changes in macrophage polarization phenotypes further intuitively (Figure 4H,K). The results showed that the number of M1 macrophages the highest in the Model group and gradually decreased in the YDC‐Gel and YDC‐Gel‐Zn groups. Conversely, the number of M2 macrophages was lowest in the model group and highest in the YDC‐Gel‐Zn group, which is consistent with the experimental results. These findings demonstrate that the YDC‐Gel‐Zn bioglue can alleviate inflammatory macrophage polarization and promote the shift of macrophages toward the anti‐inflammatory M2 phenotype by leveraging the DOPA groups in mussel‐derived peptides and grafted Zn, thereby modulating the immunophenotype of macrophages.

### 2.6 Immunomodulatory Mechanisms of the Bioglue

**Figure 5:**
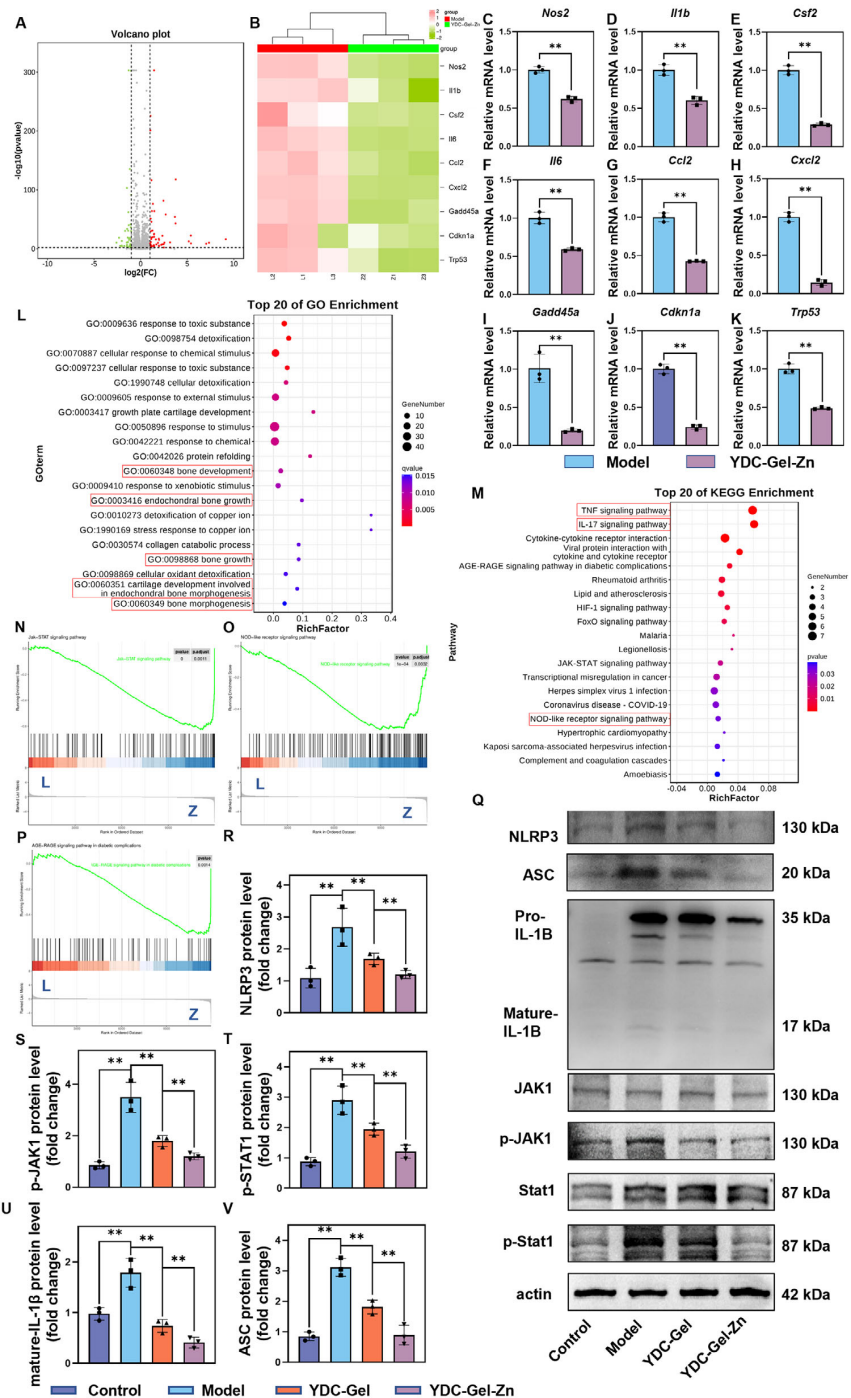
Mechanistic investigation of the immunomodulatory effects of bioglue. A) Volcano plot of differentially expressed genes (DEGs). B) Heatmap of DEGs associated with inflammation and senescence. C–K) qPCR validation of inflammation‐ and senescence‐related DEGs (*n* = 3). L) GO enrichment analysis of DEGs. M) KEGG pathway analysis of downregulated DEGs. N‒P) GSEA of inflammation‐ and senescence‐related pathways. Q–V) Western blot analysis and quantification of proteins related to the NOD‐like receptor signaling pathway and the JAK1/STAT1 signaling pathway (*n* = 3). The data are presented as the means ± SDs in the analysis figures, with *p < 0.05 and **p < 0.01 indicating statistical significance.

To further investigate the potential mechanisms and associated pathway alterations of the YDC‐Gel‐Zn bioglue in immunomodulation, we performed a high‐throughput transcriptome sequencing analysis of BMMs from the Model (L) and YDC‐Gel‐Zn (Z) groups. A principal component analysis (PCA) revealed minimal intragroup variability but significant intergroup differences, validating the reliability of the sequencing data (Figure S10A, Supporting Information). A differential gene expression analysis revealed 61 significantly upregulated and 53 significantly downregulated genes in the YDC‐Gel‐Zn group compared to those in the model group (**Figure** **5**A). Notably, the expression of the pro‐inflammatory genes (Nos2, Il1b, Csf2, Il6, Ccl2, and Cxcl2) was markedly downregulated. A qRTPCR analysis further demonstrated the pro‐inflammatory gene regulation of YDC‐Gel‐Zn, as shown by RNA sequencing, compared to the LPS group (Figure 5B–H). A gene ontology (GO) enrichment analysis revealed that five of the top 20 enriched pathways were related to bone growth, reinforcing the interconnection between immunity and osteogenesis (Figure 5L). A Kyoto Encyclopedia of Genes and Genomes (KEGG) pathway analysis of the downregulated genes revealed the involvement of inflammatory signaling pathways, including TNF, IL‐17, JAK‐STAT, and NOD‐like signaling pathways, in the immunomodulatory effects of YDC‐Gel‐Zn (Figure 5M). A gene set enrichment analysis (GSEA) confirmed the suppression of the JAK‐STAT and NOD‐like signaling pathways in the YDC‐Gel‐Zn group, consistent with the findings of the KEGG analysis (Figure 5N,P). The JAK‐STAT signaling pathway, a critical inflammatory mediator, promotes M1 macrophage polarization and inflammatory cytokine release through the activation of the JAK1‐STAT1 signaling pathway under LPS/IFN‐γ stimulation.^[^ ^42^ ^]^ Previous studies have shown a proinflammatory positive feedback loop between the JAK1‐STAT1 and NOD‐like signaling pathways; the activation of JAK1‐STAT1 induces NLRP3 expression to activate NOD‐like signaling pathways, whereas IL‐1β generated by the NOD‐like signaling pathway reciprocally enhances the activation of JAK1‐STAT1. A western blot analysis (Figure 5Q–V) demonstrated significantly elevated p‐JAK1, p‐STAT1, and NLRP3, ASC, and IL‐1β levels in the Model group. These inflammatory markers were lower in the YDC‐Gel group and further reduced in the YDC‐Gel‐Zn group to near that in control group, indicating the synergistic anti‐inflammatory effects of DOPA and Zn^2+^.

**Figure 6:**
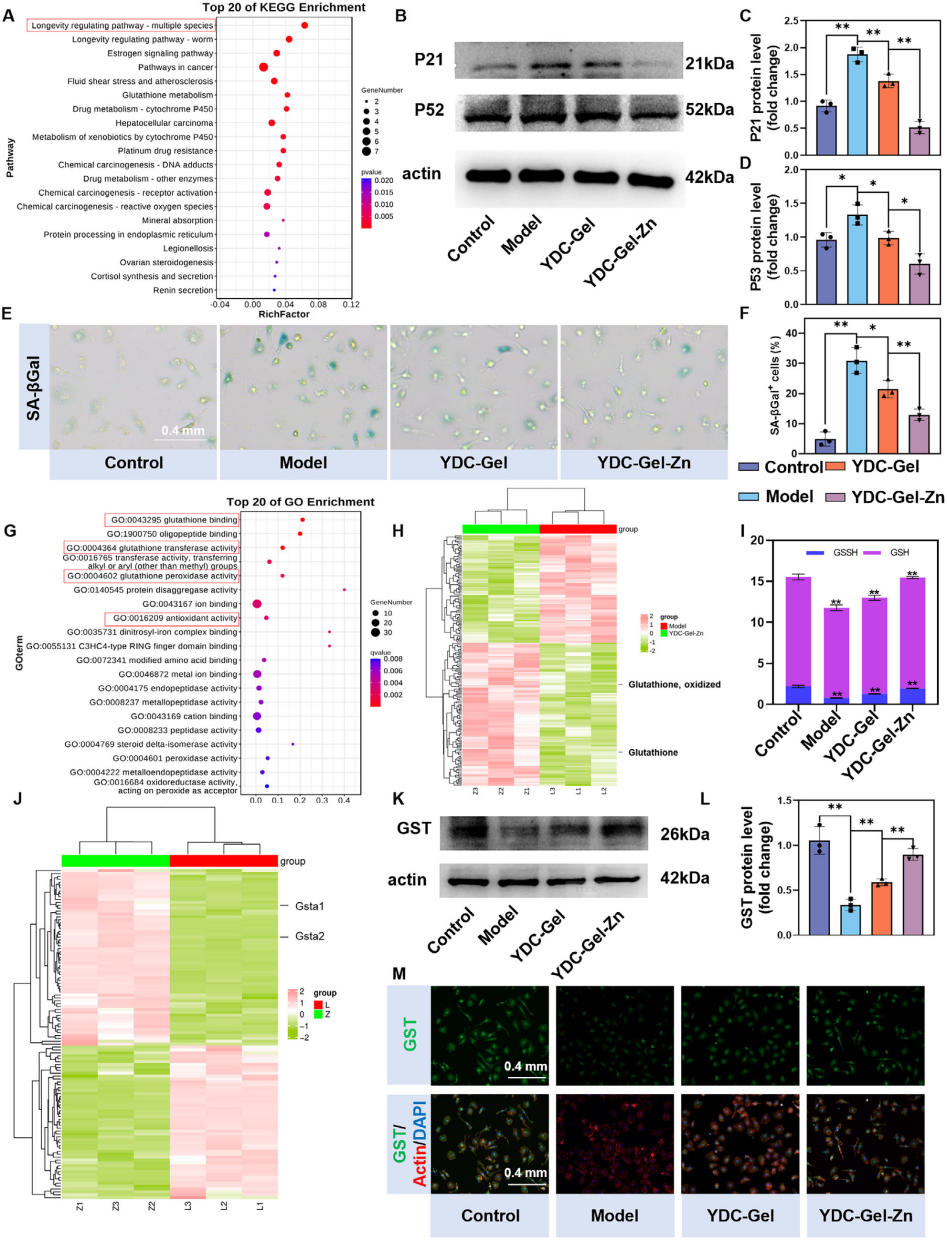
Mechanistic investigation of the role of bioglue in senescence regulation. A) KEGG pathway analysis of upregulated DEGs. B,D) Western blot analysis of senescence‐associated proteins and quantitative results (*n* = 3). E,F) Senescence‐associated β‐galactosidase (SA‐β‐gal) staining of BMMs across groups and quantitative analysis (*n* = 3). G) GO enrichment analysis of upregulated genes. H) Heatmap of differentially expressed metabolites. I) Changes in the GSSG/GSH ratio in BMMs across the experimental groups. J) Heatmap of differentially expressed genes. K,L) Western blot analysis of GST expression levels and quantification (*n* = 3). M) Immunofluorescence staining of GST expression (GST: green, Actin: red, nuclei: blue). The data are presented as the means ± SDs in the analysis figures, with *p < 0.05 and **p < 0.01 indicating statistical significance.

Next, we conducted a KEGG pathway analysis on the upregulated genes to elucidate the immunomodulatory mechanisms of the YDC‐Gel‐Zn bioglue (**Figure** **6**A). The results demonstrated significant enrichment of senescence‐associated pathways. Previous studies have shown that inflammatory cytokines induce macromolecular damage (to DNA, proteins, and lipids) in macrophages, triggering the production of senescence‐associated secretory phenotypes (SASP) and promoting the accumulation of senescent cells.^[^ ^43^, ^44^ ^]^ In contrast, M2‐polarized macrophages facilitate tissue repair and regeneration and maintain homeostasis through senescence mitigation. Transcriptome sequencing and RT‐qPCR analyses demonstrated the downregulated expression of aging‐related genes (Gadd45a, Cdkn1a and Trp53) in the YDC‐Gel‐Zn group (Figure 5B,I–K). Western blot analyses of p21 and p53 (Figure 6B–D) revealed that YDC‐Gel‐Zn substantially suppressed the expression of senescence‐related proteins in macrophages. β‐galactosidase staining (Figure 6E,F) corroborated these findings, indicating that YDC‐Gel‐Zn significantly reduced the number of senescent macrophages under inflammatory conditions. These results collectively demonstrate that YDC‐Gel‐Zn inhibits macrophage M1 polarization and senescence progression through dual suppression of the activation of the JAK1‐STAT1 and NOD‐like signaling pathways.

We subsequently performed a GO enrichment analysis on the upregulated genes to identify the protective molecular functions in the macrophages associated with the inhibition of the JAK1‐STAT1/NOD‐like signaling pathway (Figure 6G). The results revealed a significant enrichment of glutathione‐related functions, including glutathione binding (GO:00 43295) and glutathione transferase activity (GO:0 004364). Under oxidative stress, excessive ROS activates the JAK1‐STAT1 signaling pathway, exacerbating inflammation while further amplifying ROS generation, a pathogenic feedback loop. GSH, a critical endogenous antioxidant, neutralizes ROS to maintain redox homeostasis.^[^ ^45^ ^]^

To further explore the role of glutathione YDC‐Gel‐Zn in immunomodulation, we conducted a metabolomic sequencing analysis on the cells in the Model group and the YDC‐Gel‐Zn groups. Metabolomic profiling of the Model and YDC‐Gel‐Zn groups via PLS‐DA demonstrated minimal intragroup variability and distinct intergroup separation, confirming the robustness of the data (Figure S10B,C, Supporting Information). A differential analysis revealed 188 significantly altered metabolites (107 upregulated and 81 downregulated) (Figure S10D, Supporting Information). By analyzing the differentially abundant metabolites, we found that both reduced GSH and oxidized glutathione (GSSG) were upregulated in the YDC‐Gel‐Zn‐treated macrophages, suggesting increased glutathione synthesis and utilization (Figure 6H). Quantification of the GSH/GSSG ratio revealed a significant depletion of the total glutathione and GSH under inflammatory conditions (Figure 6I). Furthermore, the YDC‐Gel‐Zn treatment effectively rescued these effects. A complementary ROS flow cytometry analysis was inversely correlated with glutathione dynamics, consistent with our previous hypothesis (Figure S10E, Supporting Information). A focused analysis of glutathione cycling‐related genes revealed that Gsta2 and Gstm1, which are GST family members were the most differentially expressed genes (Figure 6J). GSTs catalyze the nucleophilic conjugation of GSH to electrophilic substrates, which is a pivotal antioxidant mechanism.^[^ ^46^ ^]^ A western blot analysis confirmed a decrease in GST expression under inflammatory conditions and YDC‐Gel‐Zn‐mediated restoration (Figure 6K,L), and an immunofluorescence analysis further validated these results (Figure 6M; Figure S10F, Supporting Information).

Next, we used a competitive inhibitor conyaining GST, S‐hexylglutathione (SHT), and the agonist of STAT1, 2‐NP, to intervene with BMM in the YDC‐Gel‐Zn treatment group to validate the crucial role of GST in immune regulation. A western blot analysis (Figure S11A–H, Supporting Information) demonstrated that in the SHT group, the expression levels of proteins related to the NOD‐like signaling pathway and M1 macrophages increased significantly. Moreover, the JAK1‐STAT1 signaling pathway was activated. However, there were no significant differences in the expression levels of the GST proteins between the treatment and the SHT groups. In the 2‐NP group, although the expression levels of proteins related to the NOD‐like signaling pathway, M1 macrophages, and p‐STAT1 increased significantly, but the expression level of p‐JAK1 increased slightly, but the expression level of GST decreased. This may be associated with the positive feedback effect of the ROS after the activation of inflammation. In an inflammatory environment, ROS accumulation can activate the JAK1‐STAT1 signaling pathway by inhibiting GST expression.

In conclusion, YDC‐Gel‐Zn promotes the antioxidant effect of glutathione by regulating the expression of GST and alleviating the accumulation of ROS in macrophages, thereby inhibiting the activation of the JAK1‐STAT1‐NLRP3 inflammatory signaling pathway and ultimately mitigating M1 polarization and senescence in macrophages.

### 2.7 Osteogenic Properties of the Bioglue

**Figure 7:**
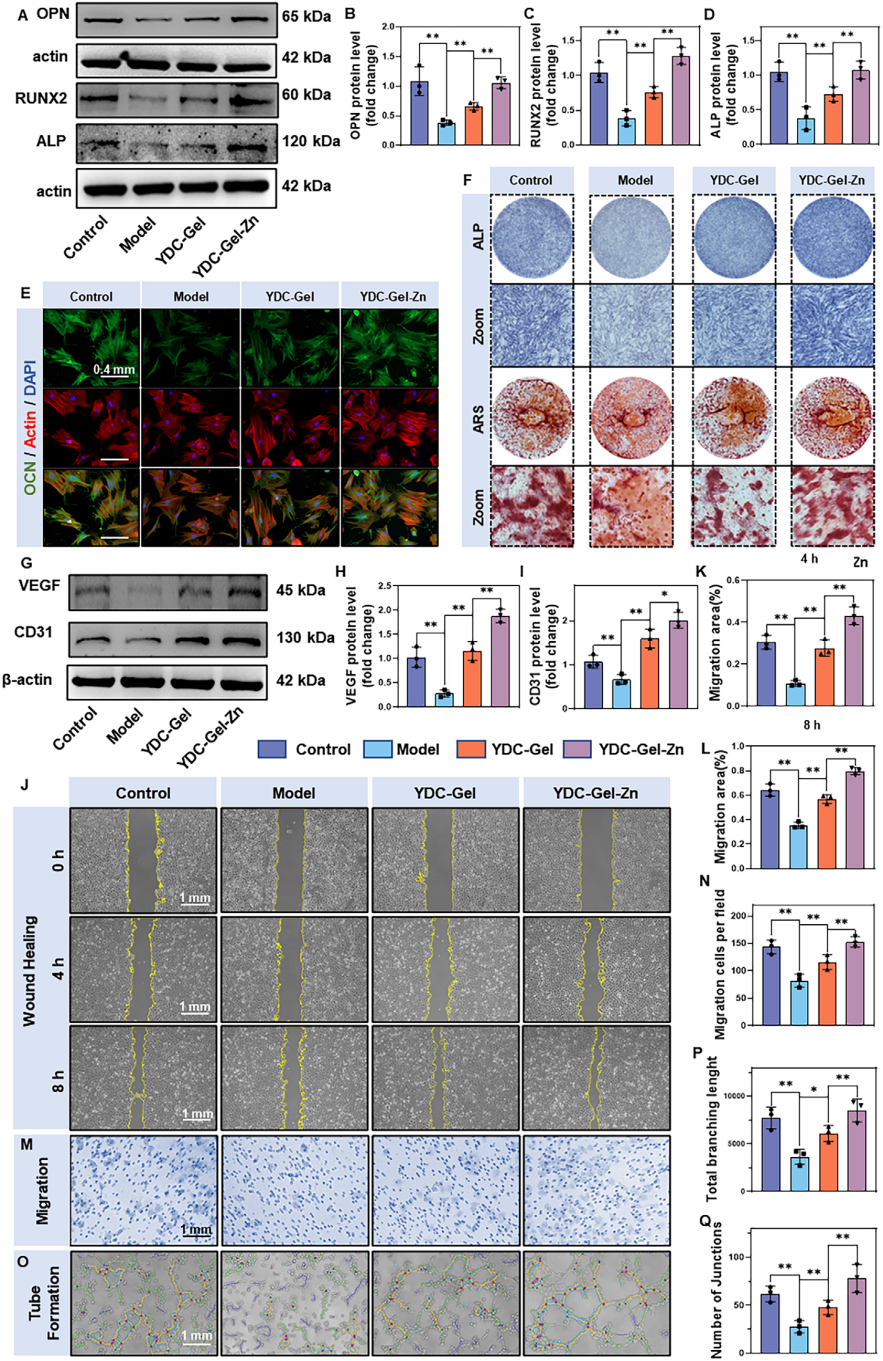
In vitro promotion of osteogenic and angiogenic differentiation. A–D) Western blot analysis of osteogenic‐related proteins (OPN, RUNX2, and ALP) in BMSCs across different groups, along with quantitative results (*n* = 3). E) Immunofluorescence staining of BMSCs to assess osteogenic differentiation (21 days, OCN: green, actin: red, nuclei: blue). F) Representative images of ALP staining (7 days) and ARS staining (21 days) in different groups. G,I) Western blot analysis of angiogenesis‐related proteins (VEGF and CD31) in HUVECs across experimental groups, with quantitative results (*n* = 3). J,L) Scratch assay was used to evaluate the migratory capacity of HUVECs in different groups, followed by quantitative analysis (*n* = 3). M,N) Transwell migration assay and corresponding quantitative analysis (*n* = 3). O,Q) Tube formation assay and quantitative assessment of angiogenic ability (*n* = 3). The data are presented as the means ± SDs in the analysis figures, with *p < 0.05 and **p < 0.01 indicating statistical significance.

The osteogenic effect of the YG peptide derived from mussels is the foundation for the ability of the bioglue to promote bone integration at the implant–bone interface. In this study, we used BMSCs to evaluate the osteogenic effects of the bioglue in vitro. We first analyzed the expression of osteogenesis‐related proteins, such as OPN, RUNX2, and ALP in the different treatment groups treated with osteogenic medium using western blotting. The results (**Figure** **7**A–D) showed that the expression of osteogenic proteins was significantly reduced in the model group, indicating that the inflammatory state was not conducive to osteogenesis. In contrast, the YDC‐Gel group showed increased protein expression compared to the Model group, suggesting that YDC can improve osteogenesis under inflammatory conditions and significantly promote bone formation. Compared to the YDC‐Gel group, the YDC‐Gel‐Zn group presented a greater increase in the expression of osteogenic proteins, further highlighting the importance of regulating the inflammatory microenvironment for osteogenesis. Immunofluorescence staining for OCN also indicated that the bioglue significantly promoted the expression of osteogenic proteins (Figure 7E; Figure S11I, Supporting Information).

We subsequently performed ALP (Alkaline Phosphatase) and ARS (Alizarin Red S) staining to assess the effect of the bioglue on extracellular matrix mineralization (Figure 7F). Compared with the inflammatory group, the YDC‐Gel group showed increased mineral deposition and calcium nodule formation under inflammatory conditions, whereas the YDC‐Gel‐Zn group exhibited significantly increased mineral deposition and calcium nodule formation, indicating that the sustained release of mussel‐derived peptides and Zn^2+^ from the bioglue promoted the osteogenic differentiation of the BMSCs. In summary, the YDC‐Gel‐Zn bioglue had excellent osteogenic capacity in vitro.

### 2.8 Angiogenesis Properties of the Bioglue

Vascular regeneration plays crucial roles in the repair and regeneration of bone tissue. Bone regeneration at the site of bone defects not only requires neovascularization to deliver nutrients and metabolic products but also relies on the vascular system to transport various regulatory factors involved in the bone integration process.^[^ ^47^ ^]^ In vitro experiments were conducted using human umbilical vein endothelial cells (HUVECs) to analyze the angiogenic effects of the proangiogenic peptides in YDC‐Gel‐Zn bioglue.

Western blotting was employed to detect CD31 and VEGF proteins, which play key roles in vascular regeneration. As shown in Figure 7G–I, the expression of CD31 and VEGF was significantly reduced under inflammatory conditions, indicating that an inflammatory environment is not conducive to vascular regeneration. This is associated with the inhibition of endothelial cell function and the release of antiangiogenic factors by inflammatory cytokines and oxidative stress. In contrast, the expressions of CD31 and VEGF were significantly higher in the YDC‐Gel group than in the Model group. This increase was related to both the CK peptide and antioxidant properties of the DOPA groups. In the YDC‐Gel‐Zn group, the expression of CD31 was slightly higher than that in the YDC‐Gel group, whereas the increase in VEGF expression was more pronounced. This may be attributed to the fact that Zn2+ not only mitigates inflammatory responses and creates a microenvironment favorable for vascular regeneration but also regulates the activity of zinc finger proteins to promote the transcription and expression of VEGF.^[^ ^48^ ^]^

Subsequently, a scratching assay (Figure 7J,L) and Transwell migration test (Figure 7M,N) were used to evaluate the effect of the bioglue on the migratory capacity of HUVECs. The scratching assay was performed at 0, 4, and 8 h, revealed that the Model group had the lowest cell migration distance. Compared with the Model group, the YDC‐Gel group showed a significantly greater cell migration distance, whereas the YDC‐Gel‐Zn group showed the strongest cell migration ability. Similarly, in the Transwell migration test, the YDC‐Gel‐Zn group exhibited the greatest number of migrated cells after 24 h of intervention. The aggregation of endothelial cells into a network structure is fundamental to neovascularization; therefore, the tube formation capacity of each group was evaluated (Figure 7O–Q). The results indicated that the bioglue groups exhibited superior tube formation capacity in the analysis of various indicators. Collectively, these results collectively suggest that the YDC‐Gel‐Zn bioglue possesses excellent angiogenic properties.

### 2.9 Immunomodulatory Effects of Bioglue In Vivo

**Figure 8:**
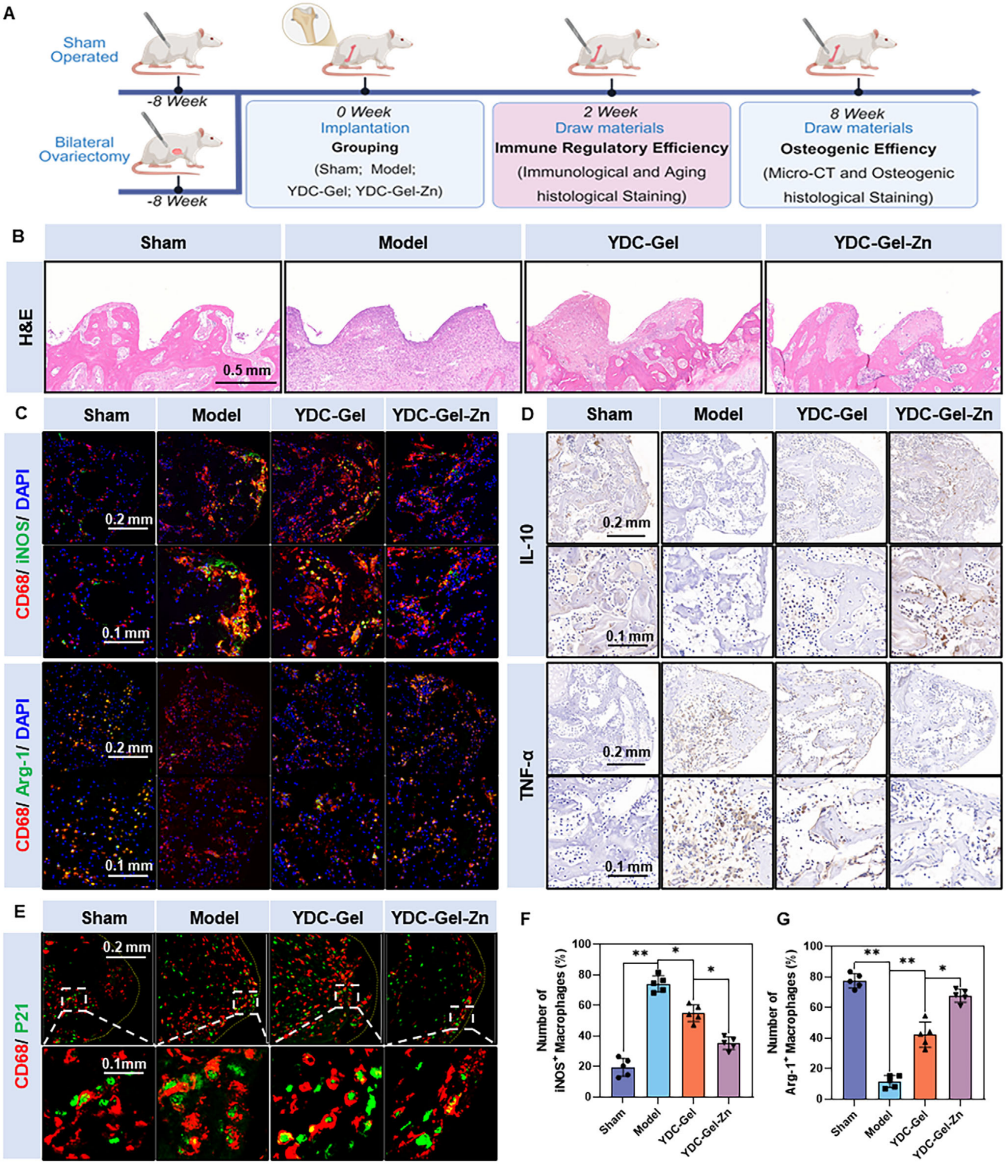
Bioglue effectively modulates the immune microenvironment at the screw‒bone interface. A) Schematic diagram of the animal experimental design (created with BioRender.com). B) H&E staining of the screw‒bone interface tissue. C) Immunofluorescence staining of macrophage infiltration at the screw‐bone interface. D) IHC analysis of the screw‒bone interface. E) Senescence‐associated immunofluorescence staining of the screw‒bone interface tissue. F,G) Proportion of iNOS^+^ and Arg‐1^+^ macrophages (iNOS^+^ & CD68^+^/Arg‐1^+^ & CD68^+^) in tissue fluorescent‐stained sections (*n* = 5). The data are presented as the means ± SDs in the analysis figures, with *p < 0.05 and **p < 0.01 indicating statistical significance.

We first established an ovariectomized (OVX) rat model of osteoporosis to verify the therapeutic effect of the bioglue in vivo (Figure S12A,B, Supporting Information). The OVX rat model represents the gold standard for osteoporosis research, demonstrating all the hallmark features of human osteoporosis, including similar patterns of bone loss, altered bone turnover markers, and compromised mechanical properties that mirror the clinical presentation of postmenopausal osteoporosis.^[^ ^49^ ^]^ The results of CT and bone parameter analyses showed that the bone quality in the OVX group was significantly lower than that in a sham group, indicating the successful establishment of the OVX osteoporosis rat model (Figure S12C,E, Supporting Information). Subsequently, screws were implanted in the distal femurs of the rats in both groups (Figure S13A,B, Supporting Information). The OVX group was further divided into three subgroups: the Model group, in which the OVX rats were only implanted with screws; the YDC‐Gel group, in which YDC‐Gel was injected into a nail hole before screw implantation; and the YDC‐Gel‐Zn group, in which the bioglue was injected. Two weeks after surgery, some rats were euthanized under anesthesia to evaluate the immune microenvironment at the bone–implant interface (**Figure** **8**A).

Because the bioglue degrades in acidic environments in vivo and biomedical materials applied in vivo must ensure that their degradation products do not exert biotoxicity on various tissues and organs, we first performed hematoxylin and eosin (H&E) on partial organs of the rats to confirm that the degradation products of the bioglue in each group caused no toxic side effects. The H&E staining results (Figure S14A, Supporting Information) demonstrated no significant pathological changes in the lungs, liver, kidneys, or spleens of the rats in any group. Subsequently, the immune microenvironment of each group was evaluated. A histopathological evaluation by H&E staining revealed distinct inflammatory profiles among the groups at a two‐week time point. The model group exhibited sustained inflammation, with substantial macrophage infiltration (Figure 8B). In contrast, both the YDC‐Gel and YDC‐Gel‐Zn groups demonstrated significantly reduced macrophage infiltration compared with the Model group. Notably, the YDC‐Gel‐Zn treatment resulted in macrophage infiltration levels comparable to those observed in the sham group. We subsequently performed immunofluorescence double staining of CD68 with iNOS or Arg‐1 in tissue sections to evaluate the immune microenvironment at the bone–implant interface. As shown in Figure 8C,F,G, the number of CD68+ macrophages was high at the bone‐implant interface in the Model group, indicating the infiltration of many macrophages. The number of CD68+ cells was slightly reduced in the YDC‐Gel group, and the number of CD68+ cells was significantly decreased in the YDC‐Gel‐Zn group. Moreover, the expression of iNOS (a marker of M1 macrophages) in CD68+ cells was high in the model group. Furthermore, there was a partial expression of iNOS in CD68+ cells in the YDC‐Gel group, whereas the expression of iNOS was significantly reduced in the YDC‐Gel‐Zn group. Conversely, the number of ARG‐1+ cells in the model group was significantly lower than that in Sham group, indicating a reduction in the number of M2 macrophages. The number of ARG‐1+ cells in the YDC‐Gel group was slightly higher than that in the model group; however, the number of ARG‐1+ cells in the YDC‐Gel‐Zn group was significantly higher.

Subsequently, we performed a histological staining analysis of the immune microenvironment at the bone–implant interface. As shown in Figure 8D and S15A–B (Supporting Information), the expression of the anti‐inflammatory factor IL‐10 was evident in the sham group, whereas its expression was significantly reduced in the model group. The expression of IL‐10 in the YDC‐Gel group was higher than that in the model group and showed a significant increase in the YDC‐Gel‐Zn group. The results of the histological staining for the inflammatory factor TNF‐a were exactly the opposite, indicating that the bone‐implant interface in an osteoporotic environment presents an inflammatory immune microenvironment and that the bioglue can significantly improve the disordered immune microenvironment. We subsequently evaluated the aging of macrophages at the bone‐implant interface (Figure 8E). The results revealed that the proportion of macrophages co‐located by CD68 and p53 in the model group was significantly greater than that in the sham group, and the number of positive cells in the YDC‐Gel group slightly decreased, while the number of positive cells in the YDC‐Gel‐Zn group decreased significantly (Figure S15C, Supporting Information), indicating that the macrophages at the bone‐implant interface significantly aged in the environment of osteoporosis, and that the YDC‐Gel‐Zn group significantly inhibited the aging of macrophages.

To assess the pro‐angiogenic efficacy of the YDC peptides in vivo, immunofluorescence staining for vascular endothelial growth factor A (VEGFA) was performed on paraffin‐embedded sections of the implant‐bone interfaces. (Figure S15E,F, Supporting Information). VEGFA expression was quantified as a surrogate marker of angiogenic activity, as it reflects dynamic endothelial activation. Compared with the sham group, the osteoporotic model group exhibited a significant reduction in VEGFA immunoreactivity, indicating severe suppression of osteogenic vascularization. Notably, the YDC‐Gel and YDC‐Gel‐Zn groups demonstrated significant increases in their VEGFA levels, with YDC‐Gel‐Zn showing superior efficacy. This reversal correlated with the immunomodulatory effects on macrophage polarization; YDC‐Gel‐Zn reduced the M1/M2 macrophage ratios in the bone microenvironment, promoting a pro‐angiogenic M2 phenotype. The polarization state of immune cells, particularly macrophages, is a central in the regulation of angiogenesis. Through immune regulation, the biological glue further enhanced the angiogenic effect of the CK peptide, significantly alleviating angiogenesis inhibition in osteoporotic environments.^[^ ^50^, ^51^ ^]^ In conclusion, the YDC‐Gel‐Zn bioglue effectively improved the disordered immune microenvironment at the bone‐implant interface in OVX osteoporotic rats, reduced macrophage M1 polarization and senescence, and promoted macrophage M2 polarization and angiogenesis.

### 2.10 Osseointegration of Bioglue In Vivo

**Figure 9:**
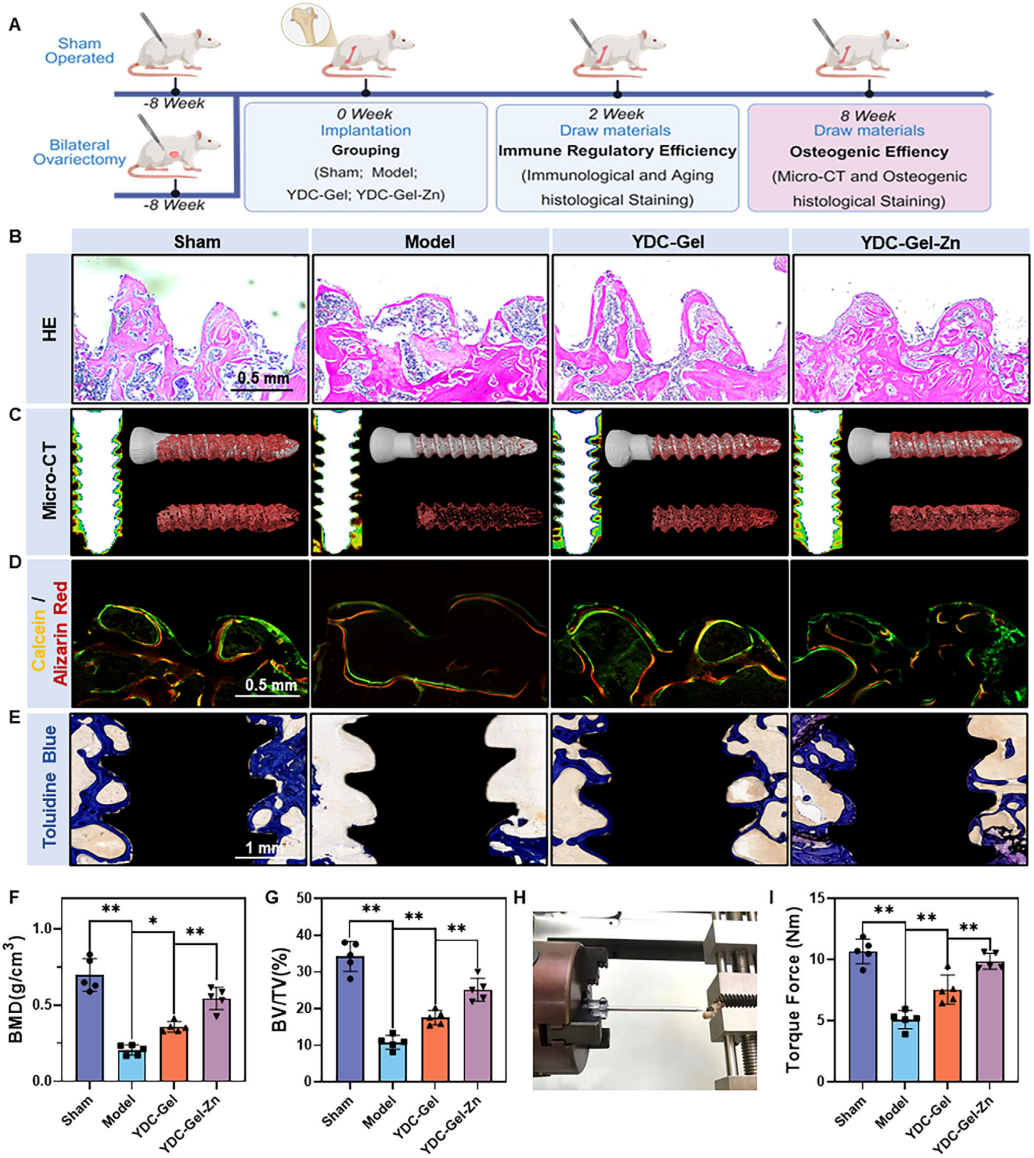
Bioglue effectively modulates the immune microenvironment at the screw‒bone interface. A) Schematic diagram of the experimental design for the animal studies (created with BioRender.com). B) Histological evaluation by H&E staining at the screw‒bone interface. C) Micro‐CT scanning and three‐dimensional reconstruction images of the implantation site. D) Double fluorescence labeling with calcein (green) and alizarin red (red) at the screw–bone interface. E) Histological evaluation by toluidine blue staining at the screw‒bone interface. F,G) Quantitative parameters of trabecular bone in each group (*n* = 5). H) Representative image of the torque‐out test. I) Quantitative analysis of the maximum extraction torque (*n* = 5). The data are presented as the means ± SDs in the figures, with *p < 0.05 and **p < 0.01 indicating statistical significance.

To verify the therapeutic effect of bioglue on bone integration in vivo, we assessed the osteointegration effect of the bioglue at the bone‐implant interface eight weeks after internal implant surgery (**Figure** **9**A). The H&E staining results revealed that the trabecular bone at the bone‐implant interface in the sham group was relatively dense at eight weeks (Figure 9B), whereas the osteoporotic model group only had an unstable ring of newly formed bone on the periphery. The osteogenic effect in the YDC‐Gel group was significantly greater than that in the YDC‐Gel group, with more regular trabecular bone formation, and the newly formed bone in the YDC‐Gel‐Zn group was even denser. Subsequently, CT was performed for each group (Figure 9C). The CT reconstruction of the newly formed bone around the screw indicated that the YDC‐Gel‐Zn bioglue could significantly enhance bone regeneration at the bone‐implant interface and osteointegration. The results of a bone parameter analysis further confirmed the CT results (Figure 9F–G).

Subsequently, we performed calcein‐alizarin red staining to assess calcium salt deposition at the bone–implant interface in each group (Figure 9D). The model group exhibited a significant reduction in calcium salt deposition, whereas the YDC‐Gel group exhibited a significant increase in calcium salt deposition; furthermore, the YDC‐Gel‐Zn group presented even greater calcium salt deposition. Toluidine blue staining showed that the area of regenerated bone at the plant surface interface in the model group was significantly smaller than that in the sham group, and the toluidine blue‐positive areas in the YDC‐Gel and YDC‐Gel‐Zn groups (particularly in the YDC‐Gel‐Zn group) were larger than those in the model group (Figure 9E; Figure S15D, Supporting Information). These results indicate that the bioglue can significantly promote bone regeneration and calcium salt deposition at the bone‐implant interface. To assess the anchoring effect of the bioglue more intuitively on implants in an osteoporotic environment, we performed a biomechanical screw‐driving test on the implanted screws in each group (Figure 9H,I). Compared with those in the sham group (10.7 ± 0.9 Nm), the screws in the osteoporotic group were more easily rotated, requiring only approximately 5.1 ± 0.7 Nm to achieve rotational displacement. The YDC‐Gel group demonstrated enhanced anchorage (7.6 ± 1.1 Nm) compared to the Model group, indicating that YDC alone exhibited robust bone‐integrating capabilities even without immunomodulatory regulation. Remarkably, the YDC‐Gel‐Zn group achieved substantially superior fixation strength (9.9 ± 0.6 Nm), recovering 93% of Sham group performance. This result not only indicates that the mussel‐derived peptides have strong osteointegration capabilities, but also demonstrates the superior stability of the bioglue with immune regulatory capabilities for internal implants in osteoporosis.

## 3 Conclusion

Through the integration of boronate‐ester bonds and metal‐phenolic coordination, we successfully synthesized a robust adhesive and injectable bioglue. This bioglue was formed via the self‐cross‐linking assembly of mussel‐derived osteogenic/angiogenic YDC peptide, Gel‐PBA, and Zn^2^⁺ ions. Currently, the primary component of clinically used medical adhesives is cyanoacrylate, which facilitates wound adhesion through interactions between polar groups.^[^ ^52^, ^53^ ^]^ However, cyanoacrylates are highly brittle, rendering them unable to withstand significant impacts and vibrations. They are commonly used for skin wound adhesion but are unsuitable for orthopedic applications. Although bone cement is currently commonly chosen in orthopedics for the fixation of implants, it is non‐degradable and only provides static frictional fixation after curing, lacking adhesive properties and biological effects. Furthermore, the implantation of bone cement is often accompanied by chronic inflammation, which can further deteriorate the microenvironment for osseointegration.^[^ ^10^ ^]^ In contrast, our synthesized YDC‐Gel‐Zn not only enables excellent dual‐interface adhesion and favorable self‐healing capabilities, but also significantly promotes osteogenic differentiation and angiogenic migration via grafted osteogenic and angiogenic peptides. YDC‐Gel‐Zn demonstrates robust adhesive capabilities in in vitro tests (137.02 ± 4.24 kPa). More importantly, in in vivo experiments, it significantly enhances osseointegration under osteoporotic conditions, with the screw torque recovering to 93% of that in normal bone at 8 weeks, demonstrating its full suitability for promoting implant osseointegration in osteoporotic environments. Moreover, the bioglue exhibited a pH‐responsive sustained release of Zn^2^⁺ ions, which, through glutathione cycle participation involving GST, significantly reduced ROS accumulation under inflammatory resolution and inhibited macrophage M1 polarization and senescence via the JAK1/STAT1/NLRP3 signaling pathway, thereby stabilizing the immune microenvironment at the bone‐implant interface. A rat model of femoral condyle implantation effectively promoted bone regeneration and interfacial stability around the implant. Benefiting from the rapid and facile self‐assembly characteristics of YDC‐Gel‐Zn, this bioglue simplifies the synthesis process, exhibiting great potential for cost‐effective mass production and clinical translation.

Nevertheless, this experiment primarily focused on the application of the bioglue in internal implant screws under osteoporotic conditions and did not deeply explore its role in complex bone‐defect environments. The application scope of bone bioglue is relatively broad, extending beyond internal implants, such as screws or pedicle screws, to include promising prospects in implantation and repair for joint replacement or massive bone defects.^[^ ^54^, ^55^ ^]^ In summary, our injectable bioglue synergistically combines the initial physical adhesion of mussel‐derived peptides, midterm immune modulation via dynamic pH responsiveness, and long‐term osteogenic and angiogenic capabilities. This approach has promising therapeutic potential for maintaining implant stability in osteoporotic conditions and has a strong clinical translational value in a wide range of application scenarios.

## 4 Experimental Section

### Preparation of Bioglue

The synthesis of YDC mussel‐derived peptide (YGFGG‐(DOPA)4‐SSSSS‐(DOPA)4‐CKKSLSLSLSLKK) was carried out via Fmoc solid‐phase synthesis by Nanjing Peptide Valley Biotech Co., Ltd. The main synthesis processes included: Select the corresponding Fmoc‐protected amino acid‐Wang Resin based on the first amino acid at the C‐terminus of the polypeptide sequence. After adding dichloromethane (DCM) to swell the resin, sequentially wash with N,N‐dimethylformamide (DMF), react with piperidine in DMF solution, then wash and dry under vacuum. Subsequently, alternately perform coupling reactions and Fmoc deprotection reactions according to the polypeptide sequence. Finally, add piperidine to DMF solution for the reaction, followed by washing and drying under vacuum. After monitoring the completion of the reaction, wash and shrink the resin to obtain the polypeptide‐resin. To cleave the peptide from the resin and simultaneously remove the protective groups from the peptide, we treated the peptide‐resin with a cleavage cocktail containing trifluoroacetic acid (TFA) as the primary component (TFA: thioanisole: H_2_O: phenol: 1,2‐ethanedithiol = 82.5: 5: 5: 5: 2.5, v/v/v/v/v). The crude peptide was then obtained by filtration and separation. Following confirmation of the correct molecular weight via mass spectrometry (MS) analysis (ZQ‐2000, Waters, USA), purify the crude peptide using HPLC (2695, Waters, USA), and then lyophilize the purified product.

### Synthesis of Gel‐PBA

First, 5 g of gelatin (Aladdin, G108397) was added to 250 mL of PBS (Cat: RG‐CE‐10,Ketu Biotech,China) and magnetically stirred at 50 °C and 500 rpm to accelerate dissolution. Five grams of 4‐carboxyphenylboronic acid (PBA, Aladdin, C101099), 10 g of N‐hydroxysuccinimide (NHS, Aladdin, D155705), and 15 g of 1‐ethyl‐(3‐dimethylaminopropyl) carbodiimide hydrochloride (EDC, Aladdin, E106172) were placed in a beaker. To this mixture, 150 mL of DMSO was added, and the mixture was magnetically stirred at 500 rpm for 2 h at room temperature to ensure complete activation. The fully dissolved DMSO solution was then slowly added to the Gel mixture, which was heated and stirred at 50 °C, and the mixture was continuously stirred at 500 rpm for 8 h to obtain the Gel‐PBA mixture. The Gel‐PBA solution was collected and transferred into a dialysis bag for dialysis in deionized water under continuous stirring. The deionized water was replaced at 3, 6, 12, and 24 h. After dialysis, the Gel‐PBA solution in the dialysis bag was collected and lyophilized for storage.

### Synthesis of YDC‐Gel‐Zn

First, 60 mg of Gel‐PBA was dissolved in 300 µL of PBS containing 2 mgmL^−1^ Zn^2^⁺ at 50 °C in a water bath. 10 mg of YDC mussel‐derived peptide was dissolved in 100 µL of DMSO at room temperature. The dissolved Gel‒PBA solution was then placed on a vortex mixer at 1000 rpm, and the YDC solution was slowly added dropwise, resulting in rapid gel formation. After freeze‐drying, the gel was dissolved in 1 mL of PBS for subsequent experiments.

### Quantitative Nuclear Magnetic Resonance (qNMR)

Using 1,3,5‐trimethoxybenzene as the reference internal standard, the mass fraction of PBA in Gel‐PBA was detected by dissolving and quantifying the H‐spectrum with deuterated DMSO. Simply put, first, the internal standard 1,3,5‐trimethoxybenzene and the Gel‐PBA to be tested were weighed and dissolved in deuterated DMSO solvent. Then, the quantitative hydrogen spectrum was collected (Bruker Avance NEO 400 MHz), and calculations were performed based on the area and molar mass of the characteristic peaks. The calculation formula is: Cx(wt.%) = (Ap/At)*(Nt/Np)*(Mp/Mt)*[mp/(mt+mp)]*100%, where Ap and At are the characteristic peak areas of 1,3,5‐trimethoxybenzene and PBA, respectively; Np and Nt were the numbers of 1H nuclei corresponding to the characteristic peaks of 1,3,5‐trimethoxybenzene and PBA, respectively; Mp and Mt were the molar masses of 1,3,5‐trimethoxybenzene and PBA, respectively; mp and mt were the weighed masses (g) of 1,3,5‐trimethoxybenzene and PBA, respectively.

### Characterization of the Bioglue

Structural Characterization: The synthesized YDC was analyzed via HPLC on an Empower system. Both YDC and Gel‐PBA were characterized via 1H NMR spectroscopy via a RX500 spectrometer (Bruker, Germany). The compositions of Gel, Gel‐PBA, YDC‐Gel, and YDC‐Gel‐Zn were examined after lyophilization via FTIR (Thermo Fisher Scientific Nicolet iS20, USA) and XPS (Thermo Scientific K‐Alpha, USA). Briefly, the prepared samples were rapidly frozen in a −80 °C freezer for 12 h. Concurrently, the freeze dryer was pre‐cooled to −50 °C, with the drying chamber maintaining a vacuum level below 10 Pa. After 3 h of continuous pre‐cooling (−50 °C, <10 Pa), the frozen samples were swiftly transferred into the freeze dryer and subjected to continuous freeze‐drying for two days. The resulting products were then sealed in an airtight container for subsequent analysis.

### Rheological Test

The rheological properties of YDC‐Gel‐Zn were assessed via a stress‐controlled rheometer (TA Instruments, UK). The YDC‐Gel‐Zn bioglue was fabricated into cylindrical specimens with a height of 2 mm and diameter of 1 cm prior to mechanical characterization. Rheological analyses were systematically conducted under controlled conditions. Strain sweep analysis was performed under a fixed frequency of 10 rads^−1^, with strain amplitude incrementally adjusted from 1% to 400% to monitor viscoelastic transitions through storage modulus (G′) and loss modulus (G″) measurements. Subsequently, frequency sweep analysis (1–100 rads^−1^ at 1% constant strain) was implemented to evaluate mechanical stability. To assess self‐healing capabilities, continuous step strain analysis (1% strain for 60 s followed by 200% strain for 60 s) was applied over 0–400 s, with continuous monitoring of G′ and G″ variations to quantify the hydrogel's recovery kinetics.

### Adhesion Characterization

Bone plates were prepared from fresh bovine femoral condyles obtained from a local butcher (Agricultural Product Market of Hefei, China) within 2 h post‐slaughter. Cortical bone sections (10 mm × 10 mm × 2 mm) were isolated from the central weight‐bearing region of the condyle using a precision bone saw. Soft tissues were removed, and the surfaces were polished with 800‐grit sandpaper to ensure uniform roughness. All plates were preserved in PBS at 4 °C and tested within 6 h of preparation. The adhesive properties of YDC‐Gel, YDC‐Gel‐Zn and CA were evaluated via an electronic universal testing machine (INSTRON, USA). Apply bioglues and CA onto the bone plate, covering an area of 6cm^2^, and then place a titanium plate on top. After fixing for three minutes, conduct the test, setting the tensile rate and shear rate to 1 and 5 mm min^−1^, respectively. The pull‐out strength of the screws was measured via a torque testing machine (HENGYI, China), after removing the femur, it was fixed in a 5% formaldehyde solution for 2 days. After being taken out and cleaned, a torque testing machine was used to measure its torque.

### Degradation of Bioglue

The bioglue was immersed in PBS at pH values of 6.8 and 7.2 and placed in a constant‐temperature incubator at 37 °C with continuous gentle shaking (80 rpm). At predetermined time points, the bioglue was removed, and surface moisture was carefully wiped off before its weight was recorded. The remaining weight percentage of the hydrogel was calculated as follows:

The remaining weight ratio (%) = (Wt/W0) × 100%, where Wt represents the weight at time t and W0 was the initial weight.

### SEM and Mapping

The morphology and surface elemental distribution of Gel, Gel‐PBA, YDC‐Gel, and YDC‐Gel‐Zn were observed using scanning electron microscopy (SEM; ZEISS Sigma 300, Germany). Freeze‐dried samples were fractured using liquid nitrogen to expose their cross‐sections, followed by gold sputtering to enhance conductivity. Samples were then imaged in the low‐vacuum chamber of the SEM. SEM mapping scans were performed simultaneously with SEM imaging: the energy‐dispersive X‐ray spectroscopy (EDS) detector coupled to the SEM synchronously recorded X‐ray spectra for each pixel, automatically identified spectral peaks, and ultimately generated 2D color distribution maps.

### BMSCs Isolation and Expansion

Female SD rat (200 ± 20 g, 8 weeks old) femurs were harvested under aseptic conditions through surgical dissection. Marrow tissue was flushed from intramedullary canals with α‐MEM, followed by erythrocyte lysis (Beyotime C3702) and centrifugation (1500 ×g). Pelleted cells were cultured in complete medium (α‐MEM + 10% FBS + 1% P/S) under standard conditions (37 °C, 5% CO_2_), with the medium replaced every 48 h. Third‐passage cultures exhibiting 80% confluency were selected for downstream applications. Primary BMSCs from 5 rats were pooled after the first passage to reduce biological variability and expand cell numbers for subsequent experiments. Using flow cytometry to identify membrane surface marker proteins of primary cells, cells that were CD45 (Biolegend, Cat:202 207) negative and CD90 (Biolegend, Cat:202 515) positive can be considered as BMSCs.

### BMMs Isolation and Differentiation

C57BL/6J murine femurs (15 ± 5 g, 5 weeks old) were aseptically excised and dissected. The marrow was lavaged with PBS, followed by erythrocyte lysis and centrifugation (1500 ×g). Pelleted cells were cultured in complete medium (α‐MEM + 10% FBS + 1% P/S) supplemented with macrophage colony stimulating factor (M‐CSF, 30 ng ml^−1^) under standard conditions (37 °C, 5% CO_2_). Differential medium management included partial medium exchange (day 3). The medium was completely renewed after nonadherent cell removal (day 6). Tightly adherent populations were confirmed as mature BMMs. BMMs from five mice were pooled after 6 days of M‐CSF differentiation to ensure a consistent macrophage population for in vitro assays. Using flow cytometry to identify membrane surface marker proteins of primary cells, cells that are double positive for CD68 (Biolegend, Cat:137 005) and CD11b (Biolegend, Cat:201 809) can be considered as BMMs.

### Zn^2+^ and YDC Release Behavior

Bioglue samples were immersed in 1 mL of PBS for physiological simulation (37 °C, 80 rpm orbital shaking). Scheduled sampling involved 50% medium replacement with preequilibrated PBS at defined intervals. The collected aliquots were cryopreserved (−20 °C) prior to analytical processing. The zinc ion concentration in the dissolved solution was detected using inductively coupled plasma optical emission spectrometry (ICP‐OES). Briefly, nitric acid was first added to the test solution, followed by low‐temperature heating. The solution was then filtered through a 0.45 µm filter membrane to remove undissolved particles and eliminate peptides. Standard solutions of zinc ions (0, 1, 5, 10, 20 mg L^−1^) were prepared using nitric acid, and their signal intensities were measured. A linear equation (Y = kX + b) was fitted with signal intensity (Y) against standard solution concentration (X), where R^2^ ≥ 0.99. The test solution was then analyzed using the ICP‐OES, and the zinc ion concentration in the sample was calculated based on the measured signal intensity and the standard curve. For YDC peptide concentration detection, high‐performance liquid chromatography (HPLC) was employed. First, gelatin potentially present in the dissolved solution was precipitated using trifluoroacetic acid (TFA). After centrifugation and filtration, ethylenediaminetetraacetic acid (EDTA) was added, and the solution was further filtered through a 0.45 µm aqueous filter membrane. A stock solution of the synthesized high‐purity YDC peptide was prepared by dissolving it in a TFA aqueous solution and subjected to gradient dilution. Separation was performed using a reverse‐phase C18 column with a column temperature set at 40 °C. The absorption peak of the standard solution at 220 nm was detected, and a linear equation (Y = kX + b) was fitted with peak area (Y) against concentration (X), where R^2^ ≥ 0.99. The processed test solutions were then analyzed, and the polypeptide concentration in the samples was calculated by substituting their peak areas into the established equation.

### Biocompatibility Assessment

Cell Proliferation: BMMs/BMSCs (2 × 10⁴ cells/well, 24‐well plate) were cultured with PBS and three concentrations of YDC‐Gel‐Zn bioglue extraction solutions (0.33, 0.66, and 1.32 µM) for 1/3/5 days. Metabolic activity was quantified via a CCK‐8 assay (Beyotime C0037) at a wavelength of 450 nm. Live/dead cell staining: Parallel cultures (5 × 10⁴ cells/well) were stained via a Calcein/PI Cell Viability (Cat: RG‐HX‐01, Ketu Biotech, China) posttreatment. The live/dead cell distribution was documented through fluorescence microscopy.

### Hemolysis Assay

Female SD rat (200 ± 20 g, 8 weeks old) erythrocytes were isolated through PBS dilution‒gradient centrifugation (3 min), and the RBCs were treated with different interventions and centrifuged for 3 min. Experimental groups were assessed against established controls: negative control: 0.9% NaCl (0% hemolysis); positive control: ddH_2_O (100% hemolysis), 1.32 µm YDC‐Gel group, and 1.32 µm YDC‐Gel‐Zn group. Posttreatment samples underwent macroscopic documentation postcentrifugation and spectrophotometric quantification of the supernatant at 580 nm.

Hemolysis rate (%) = (Absorbance of the experimental group–Absorbance of the negative control)/(Absorbance of the positive control–Absorbance of the negative control) × 100%.

### In Vivo Hemostasis Assay

Female SD rat (200 ± 20 g, 8 weeks old) were intraperitoneally injected with heparin (3000 U kg^−1^) 30 min before surgery to achieve anticoagulation. The rats were anesthetized with sodium pentobarbital and fixed on a surgical board. The filter paper was weighed, and the rat liver was exposed through an abdominal incision. A portion of the liver edge was removed and placed on filter paper. A longitudinal incision was made at the distal boundary of the liver, and the prepared medical hemostatic sponge (Absorbable Gelatin Sponge, Xiangen, China), YDC‐Gel, and YDC‐Gel‐Zn were applied to the incision site. Images of the bleeding were captured at 30 and 60 s, after which the filter paper was weighed.

### In Vitro Coagulation Assay

Whole blood was collected from the rats, and 100 µL of fresh whole blood was pipetted onto a six‐well plate, medical ddH_2_O (Hemolysis Group), PBS (Control), hemostatic sponge, 1.32 µm YDC‐Gel, and 1.32 µm YDC‐Gel‐Zn. Meanwhile, 100 µL of whole blood was diluted in PBS and centrifuged. After repeating this process five times, red blood cells were obtained and added to the well plate as a negative control group. The samples were incubated at 37 °C for 10 and 30 min. After incubation, 2 mL of ddH_2_O was added, and the samples were placed on a shaker at 80 rpm for 3 min at room temperature. Macroscopic images were captured, and the absorbance of ddH_2_O at 580 nm was measured.

### Cell Treatment

Different cellular models were used in this study according to the specific aims of each experimental part. BMMs were isolated from C57BL/six mice and used for experiments related to immunoregulation and sequencing. BMSCs were harvested from the same mouse strain to investigate osteogenic differentiation. For the angiogenesis studies, HUVECs were purchased and cultured following standard protocols. Detailed descriptions of the isolation, culture, and treatment for each cell type were provided in their respective sub‐sections below. In the in vitro experiment, cells were divided into four groups: control group, model group, YDC‐Gel extract group, and YDC‐Gel‐Zn extract group. All groups except the control were first exposed to 500 ng mL^−1^ LPS in complete culture media (α‐MEM with 10% FBS for BMMs/BMSCs; ECM with 10% FBS for HUVECs) for 12 h. The YDC‐Gel and YDC‐Gel‐Zn groups were first treated with 500 ng mL^−1^ LPS for 12 h, followed by culture in medium containing 1.32 µM YDC‐Gel or YDC‐Gel‐Zn extract for an additional 24 h, respectively. All cells were cultured in a 37 °C incubator containing 5% CO2.

### Western Blot

Cells were harvested after treatment and solubilized in RIPA buffer to obtain total protein extracts. The concentrations of protein in each group were standardized via a BCA protein assay (Thermo Fisher Scientific) with normalization to equal loading volumes. The proteins were separated via SDS‒PAGE, followed by electrotransfer to nitrocellulose membranes. Membrane blocking was performed via the use of QuickBlock Protein‐Free Blocking Buffer for Western Blot (Beyotime, P0240) for 0.5 h at ambient temperature. Primary antibody binding overnight at 4 °C. β‐actin (AC026): 1:10 000, NLRP3 (A24294): 1:1000, ASC (A22046): 1:1000, IL‐1B (A16288): 1:1000,P21 (A19094): 1:1000, P53 (A19585): 1:1000, GST (A1628): 1:1000, OPN (A1361): 1:1000, RUNX2 (A11753): 1:1000, ALP (A0514): 1:1000, VEGF (A23759): 1:1000, CD31 (A19014): 1:1000, were purchased from ABclonal (China), JAK1 (AF5012): 1:1000, p‐JAK1 (AF2012): 1:1000, STAT1 (AF6300): 1:1000, p‐STAT1 (AF3300): 1:1000, were purchased from Affinity (USA). After primary incubation, the membranes were washed three times with TBST washes (5 min each) prior to secondary antibody (ABclonal, AS014) exposure. Horseradish peroxidase‐conjugated species‐specific secondary antibodies were applied for 60 min. Quantitative densitometric analysis was performed via enhanced chemiluminescence detection (Monad, China) followed by digital image processing with ImageJ software (NIH). All the data were normalized to those of β‐actin controls for comparative expression analysis.

### Quantitative Real‐Time PCR (RT‐PCR)

Total RNA was extracted from the cellular samples via TRIzol reagent (Beyotime, R0016, China), after which the total RNA was quantified with a NanoDrop 2000 spectrophotometer (Thermo Fisher Scientific). A standardized quantity of 1 µg of RNA was reverse transcribed to generate complementary DNA (cDNA) via Multiplex One Step RT‐PCR Kit (Yeasen,China). Subsequent amplification was conducted via the CFX96 Real‐Time PCR System (Bio‐Rad Laboratories, USA), with the primer sequences provided in Table S1 (Supporting Information).

### Immunofluorescence Staining

Cell cultures were established on 24‐well plates with coverslips at the bottom. After treatment, the culture medium was removed, and the cells were fixed in 4% paraformaldehyde (10 min). Sequential processing included Triton X‐100 permeabilization (0.1%, 15 min) and immunofluorescence blocking buffer (Beyotime, P0102, China, 30 min) exposure at room temperature. Primary antibody binding occurred overnight at 4 °C, followed by three rinses with PBS. The secondary antibody (ABclonal, AS053 and AS054) incubation utilized species‐specific fluorescent conjugates (1 h, 37 °C, dark conditions). Antifade Mounting Medium with DAPI (Beyotime, P0131) was used before image acquisition, and fluorescence microscopy imaging was performed on Zeiss inverted systems. Quantitative fluorescence analysis was performed via ImageJ software.

### Flow Cytometry Analysis

After the cell treatment was completed, the medium was aspirated and discarded. The cells were washed with PBS, followed by the addition of trypsin (Yeasen, 40126ES60, China) to detach the cells. The digestion was terminated by adding serum‐containing medium. After centrifugation, the supernatant was aspirated and discarded. The cells were then washed with PBS and centrifuged again. Finally, a single‐cell suspension was prepared by resuspending the cell pellet in PBS. The cells were incubated with flow cytometry antibodies, CD11b (Biolegend, Cat: 101 211), CD86 (Biolegend, Cat: 159 203) and CD206 (Biolegend, Cat: 141 705), followed by washing with PBS. The samples were analyzed via flow cytometry (BD FACSCanto II, USA). The quantitative immunophenotyping data were generated via FlowJo 10 software (Tree Star, USA).

### Osteogenic Induction and Identification

BMSCs were plated at 2 × 10⁴ cells/well density in 24‐well culture plates. Osteogenesis was stimulated via α‐MEM supplemented with 10% FBS, 10 mM β‐glycerophosphate, 50 µM ascorbic acid, and 100 nM dexamethasone under specified experimental conditions. At 7 days postinduction, cellular ALP expression was evaluated through three steps: formaldehyde fixation (4%), membrane permeabilization (0.1% Triton X‐100), and chromogenic visualization via BCIP/NBT detection reagents (Solarbio, China). Quantitative ALP activity measurement was performed via a standardized enzymatic assay kit (Solarbio, China) per the manufacturer's specifications. Mineralization assessment was conducted at 21 days through formaldehyde fixation (4% paraformaldehyde), PBS rinsing, and Alizarin Red S staining (2%, Solarbio, China), followed by a 30‐min incubation at ambient temperature. Unbound dye removal was achieved through distilled water irrigation, with mineralized nodules visualized under light microscopy. Calcium quantification was executed via cetylpyridinium chloride‐mediated nodule solubilization (10% w v^−1^), with the absorbance measured at 562 nm using microplate spectrophotometry.

### Scratch Assay

HUVECs (National Collection of Authenticated Cell Cultures) were seeded in six‐well plates in medium supplemented with 2% FBS. When the cell confluence reached 90%, a straight scratch was made through the center of the wells via a 200 µL pipette tip. The detached cells were washed away with PBS, treated with different interventions and cultured at 37 °C in a 5% CO_2_ incubator. Cell migration was observed and photographed at 0, 4, and 8 h via an inverted optical microscope. Fiji software was used to analyze the cell migration images.

### Angiogenesis Assay

GelNest Matrigel (NEST Biotechnology, China) stored at −20 °C was thawed overnight at 4 °C. Precooled 24‐well plates and 200 µL pipette tips were prepared. The thawed Matrigel was mixed with precooled tips and evenly distributed into the wells. The plates were incubated at 37 °C for 1 h to allow the Matrigel to solidify. HUVECs were seeded at a density of 8 × 10⁴ cells per well in 500 µL of cell suspension in medium supplemented with 2% FBS, and each group was treated accordingly. The plates were incubated in a cell culture incubator, and angiogenesis was observed and photographed at 6 h via an optical microscope. Fiji software was used to analyze angiogenesis parameters.

### Transwell Assay

Cell populations were plated onto 12‐well Transwell inserts (8 µm pore size) and maintained in serum‐deprived medium (0% FBS, 500 µL) for 12 h. The lower chamber contained medium with 10% FBS as a chemoattractant, LPS was added in Model group, YDC‐Gel and YDC‐Gel‐Zn were based on Model group with the addition of YDC‐Gel or YDC‐Gel‐Zn at 1.32 µM. Cultures were maintained under standard conditions (37 °C, 5% CO_2_) for 12 h. Post‐incubation, inserts were transferred to new wells containing fixative (4% paraformaldehyde) and incubated at 37 °C for 30 min. Cellular debris was removed using PBS‐moistened cotton applicators. Specimens underwent three PBS rinses prior to crystal violet staining (5% w v^−1^). Visual documentation of migratory cells was performed using upright light microscopy, with representative images acquired from membrane undersides.

### RNA Sequencing

Total RNA from BMMs was collected and purified using TRIzol extraction reagent (Invitrogen, #15596018CN). Sequencing was performed by Shanghai Yuanxin Biotechnology Co., Ltd. Strand‐specific libraries for transcriptome sequencing were constructed using the TruSeqTM RNA Sample Prep Kit (Illumina). The libraries were then sequenced on the HiSeq 2000 TruSeq SBS Kit v3‐HS (Illumina) platform. Differentially expressed genes (DEGs) were identified using DESeq2 software (R Package, version 4.1.0). P values were adjusted for multiple hypothesis testing via the Benjamini and Hochberg method. A corrected P value < 0.05 and an absolute log2(fold change) ≥ 0.5 were set as the thresholds for significant differential expression.

### Bilateral OVX Model and Femoral Screw Implantation Model

Female Sprague‐Dawley (SD) rats (200 ± 20 g), 8 weeks old, ten rats in each group, totaling 40 rats, were acquired from the Animal Center of Anhui Provincial Hospital (Hefei, China). Prior to surgery, all the animals were maintained under specific pathogen‐free (SPF) conditions for 1 week with ad libitum access to food and water. All experimental procedures were ethically approved by the Ethics Committee of the First Affiliated Hospital of University of Science and Technology of China (approval number: 2024‐NA‐0255).

OVX or sham surgery was conducted following standardized protocols. Under sterile conditions, rat dorsa were disinfected with iodine and medical alcohol, and animals were positioned prone. After dorsal skin incision, bilateral ovaries were excised via uterine tube ligation. In sham‐operated rats, surrounding ovarian adipose tissue was resected analogously. One month post‐surgical intervention, osteoporosis model establishment was confirmed using micro‐CT and histological analysis. Upon successful model induction, rats were randomly allocated into four groups. A femoral screw implantation model was then developed: a vertical incision was created along the medial knee joint to expose the distal femur; a drill hole was made through the femoral condyle, traversing both cortical layers; the prepared hole was prefilled with 200 µL of Gel, YDC‐Gel, or YDC‐Gel‐Zn, all filling materials were sterilized under a 254 nm UV light for 40 min; and titanium screws (the shank length was ≈6 mm. The threaded bore diameter was 2 mm, and the screw tip features a 90° conical angle) were bilaterally implanted into the femoral condyles. The surgical incisions were closed using layered suturing. Euthanasia was performed at 2 and 8 weeks post‐surgery, with subsequent femur collection. Additionally, rats euthanized at week 8 after surgery received intramuscular injections of 20 mg k^−1^g calcein and 30 mg k^−1^g alizarin red at 21 days and 7 days prior to euthanasia.

### Micro‐CT Analysis

Femurs fixed in 4% paraformaldehyde for 24 h were transferred to 70% ethanol. Fixed femurs were scanned via micro‐CT (µCT 80, Scanco Medical; the resolution was 18 µm, the voltage was 50 kV, the current was 500 µA, and the exposure time of each image was 100 ms). The acquired data were reconstructed into 2D images via NRecon software. The region of interest (ROI) at the distal femur was selected via DataView software, and 3D reconstruction and analysis of bone parameters, including bone mineral density (BMD, mg cm^−^^3^), were performed via CTAn software. 3D images were rendered and visualized via Mimics Medical software.

### Histological Staining

H&E Staining: After micro‐CT scanning, all the histological tissues were collected and fixed in 4% paraformaldehyde for at least 48 h, followed by decalcification in 10% ethylenediaminetetraacetic acid (EDTA) for one month, EDTA was changed every two days. Following the melting of the embedding medium, tissue sections undergo a deparaffinization and rehydration process. Sections were then stained with hematoxylin solution (Solarbio, G4520) for 2 min, rinsed with water, and differentiated in 1% hydrochloric acid ethanol for 10–15 s, followed by a water rinse. Subsequently, sections were treated with 1% ammonia water for 30 s, washed again, and counterstained with 1% eosin Y solution for 3 min. Dehydration was carried out through graded ethanol solutions (90%, 95%, and 100%, each for 3 min), and sections were cleared in xylene (3 min × 2). Finally, slides were air‐dried in a fume hood and permanently mounted using neutral resin and coverslips. Images were captured using an upright microscope (Zeiss, Germany).

### Immunohistochemistry and Immunofluorescence Staining

Tissue sections were deparaffinized and rehydrated, followed by antigen retrieval via 0.15% trypsin solution. Sections were incubated with primary antibodies, iNOS (ABclonal, A3774), Arg‐1 (ABclonal, A25808), CD68 (Proteintech, 3A9A7), P21 (ABclonal, A19094), VEGF (ABclonal, A19094), IL‐10 (ABclonal, A2171), TNF‐α (ABclonal, A11534), at 4 °C overnight. For immunofluorescence staining, after incubation with fluorescent secondary antibodies, (ABclonal, AS053, AS054), the nuclei were stained with DAPI. Sections were dehydrated and mounted with neutral resin, and images were captured using an inverted fluorescence microscope. For immunohistochemistry, deparaffinization and rehydration were performed as described in H&E staining. Tissue sections were then incubated with 2 mg mL hyaluronidase for 70 min, followed by 2 mg mL^−1^ protease for 10 min, with three 5‐min washes in enzyme‐free water. After blocking with horse serum for 30 min, sections were incubated with pre‐diluted primary antibody overnight at 4 °C. Subsequent steps included three 5‐min PBS washes, 60‐min incubation with secondary antibody (ABclonal, AS014) at room temperature, and another three 5‐min PBS washes. The signal was amplified using SAB complex for 60 min, followed by three 5‐min PBS washes. DAB substrate was applied for color development, with reaction time monitored under the microscope, and sections were washed with deionized water. Hematoxylin counterstaining was performed for 2 min, followed by 10–15 s of differentiation in 1% hydrochloric acid ethanol, 30 s in 1% ammonia water, and three 5‐min ethanol dehydration steps (90%, 95%, 100%). Finally, slides were cleared in xylene, air‐dried, and mounted with resin for microscopic examination and imaging (Zeiss, Germany). Quantitative analysis was performed via ImageJ software.

### β‐Galactosidase Staining

Staining solution was prepared according to the manufacturer's instructions (Beyotime, C0602). Cells grown on coverslips were fixed with 4% paraformaldehyde and immersed in the staining solution overnight at 37 °C. After the staining solution was removed, images were captured via an upright microscope.

### Statistical Analysis

All the data were presented as the means ± standard deviations (SDs). Statistical analyses were performed via GraphPad Prism v.8.0. The t test was used to analyze differences between two groups. For comparisons among more than two groups, one‐way ANOVA was used to identify significant differences, followed by Tukey's post hoc test for multiple comparisons. Error bars in the figures represent standard deviations, and a probability value (P) < 0.05 was considered statistically significant.

## Conflict of Interest

The authors declare no conflict of interest.

## Supporting information



Supporting Information

## Data Availability

The data that support the findings of this study are available from the corresponding author upon reasonable request.
