# Supplementary material for: A Pseudo‐Mytilus Edulis Foot Protein‐Based Hydrogel Adhesive with Osteo‐Vascular‐Immune Coupling Effects for Osteoporotic Bone‐Implant Integration
Source: Adv Mater. 2025 Nov 5;38(6):e11840. doi: 10.1002/adma.202511840 (PMC12848646; doi:10.1002/adma.202511840)
Supplement: Supplementary file 1 — Supporting Information [file ADMA-38-e11840-s001.docx]

**Supplementary Materials**

**A Pseudo-Mytilus Edulis Foot Protein-Based Hydrogel Adhesive with Osteo-Vascular-Immune Coupling Effects for Osteoporotic Bone-Implant Integration**

*Wentao Wang, Zhenyu Li, Siming Zhang, Yue Ma***, Lei Yu, Qidong Zhang, Guoqing Pan, Dechun Geng***, Chen Zhu***, Jiaxiang Bai**

Dr. W. Wang, Dr. Z. Li, Dr. S. Zhang, Prof. Dr. C. Zhu, Prof. Dr. J. Bai

Department of Orthopedics, Centre for Leading Medicine and Advanced Technologies of IHM, The First Affiliated Hospital of USTC, Division of Life Sciences and Medicine, University of Science and Technology of China, Hefei, 230022, Anhui, China.

E-mail: [jxbai1995@ustc.edu.cn](mailto:jxbai1995@ustc.edu.cn) (J. Bai), [zhuchena@ustc.edu.cn](mailto:zhuchena@ustc.edu.cn) (C Zhu)

Dr. W. Wang, Prof. D. Geng

Department of Orthopedics, The First Affiliated Hospital of Soochow University, 188 Shizi Street, Suzhou 215006, Jiangsu, China.

E-mail: [szgengdc@suda.edu.cn](mailto:szgengdc@suda.edu.cn) (D. Geng)

Prof. Y. Ma, Prof. G. Pan

School of Chemistry and Chemical Engineering, Jiangsu University, 301 Xuefu Rd, Zhenjiang, 212013, Jiangsu, China.

E-mail: [yamma@ujs.edu.cn](mailto:yamma@ujs.edu.cn) (Y. Ma)

Dr. W. Wang

Department of Orthopedics, Peking University First Hospital, Beijing, People's Republic of China.

Dr. L. Yu

Department of Orthopedics, Qilu Hospital of Shangdong University, Shandong University, Jinan, 250100, Shandong, China.

Prof. Dr. Q. Zhang

Department of Orthopeadics, China-Japan Friendship Hospital, Beijing, 100029, China.


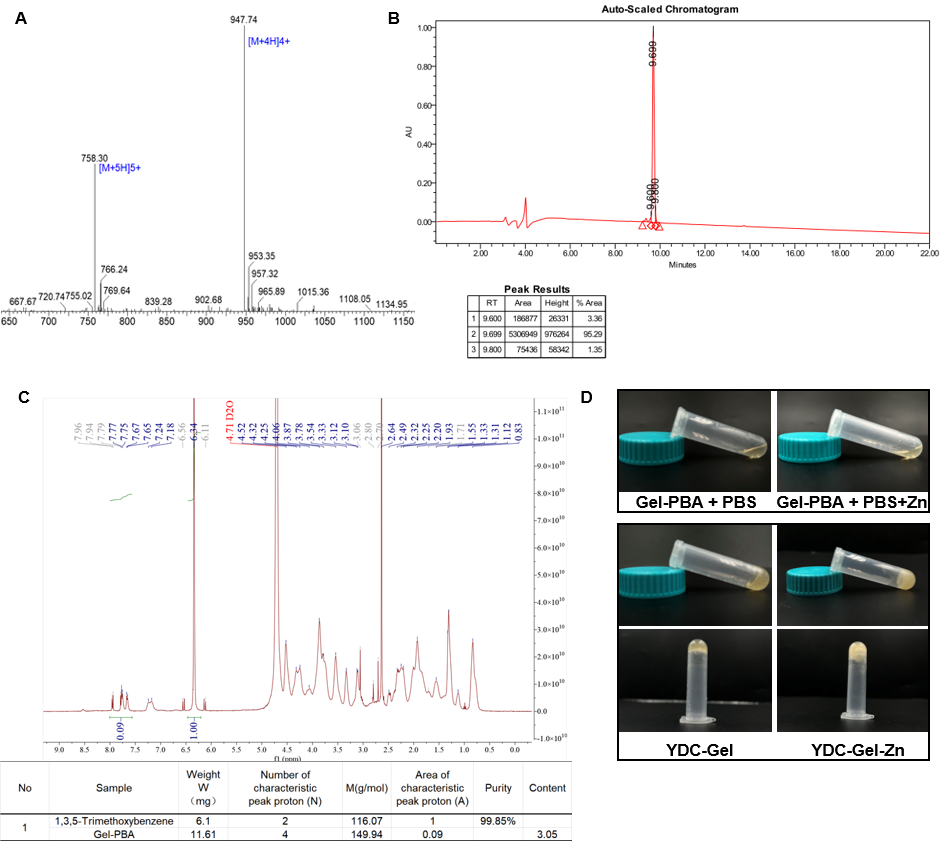


**Figure S1. A**) ESI‒MS analysis results for the YDC peptide. **B**) HPLC analysis of the YDC peptide. **C**) qNMR of Gel-PBA. (**D**) Macroscopic appearance of YDC-Gel and YDC-Gel-Zn bioglue.

**
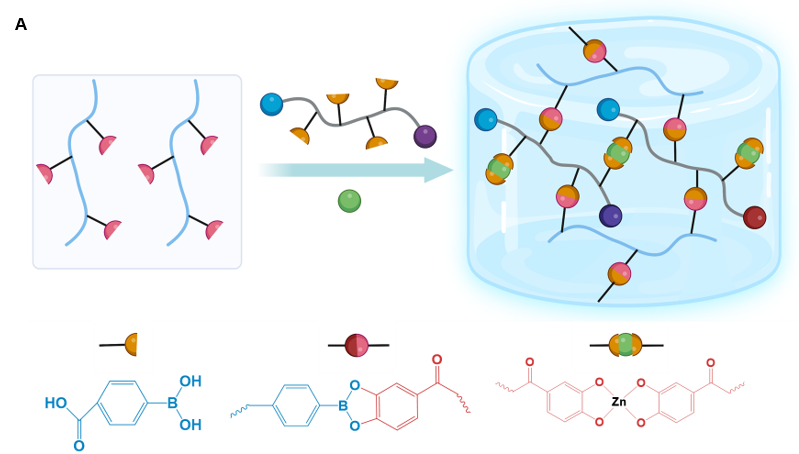
**

**Figure S2. A**) Schematic representation of bioglue formation through boronate ester bonds and metal‒phenolic coordination between YDC, Gel‒PBA, and Zn²⁺ (created with BioRender.com).

**
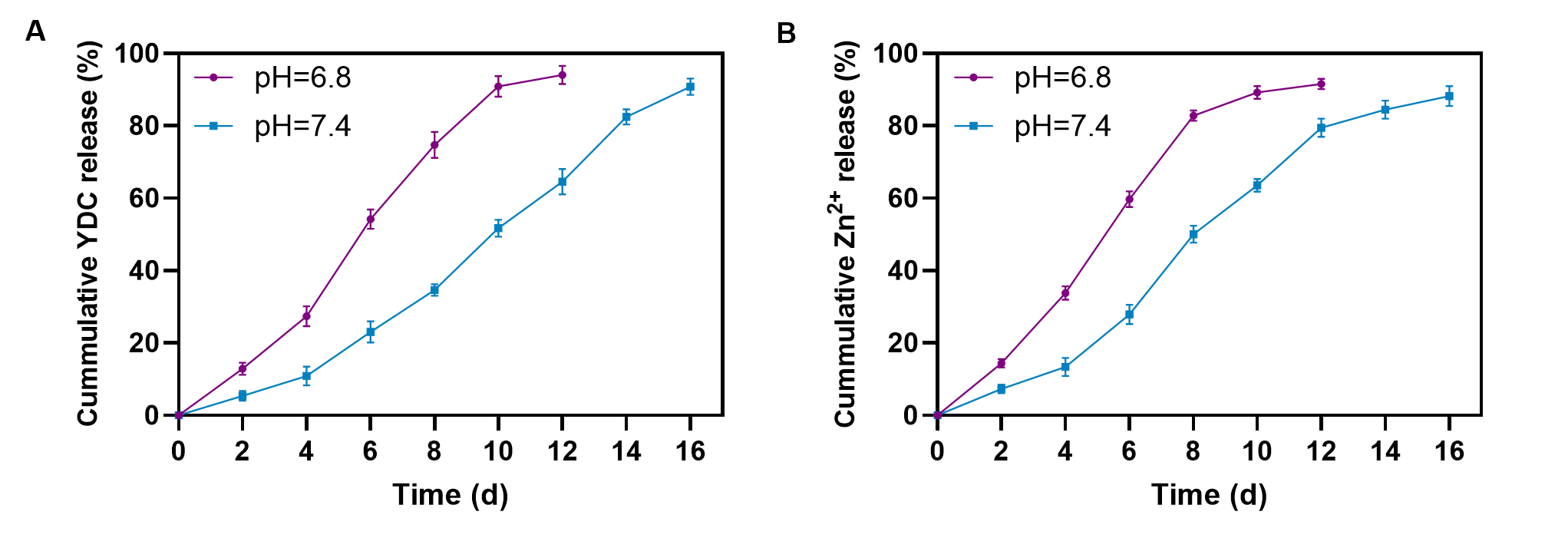
**

**Figure S3. A**) Release curve of YDC peptide. **B**) Release curve of Zn^2+^.


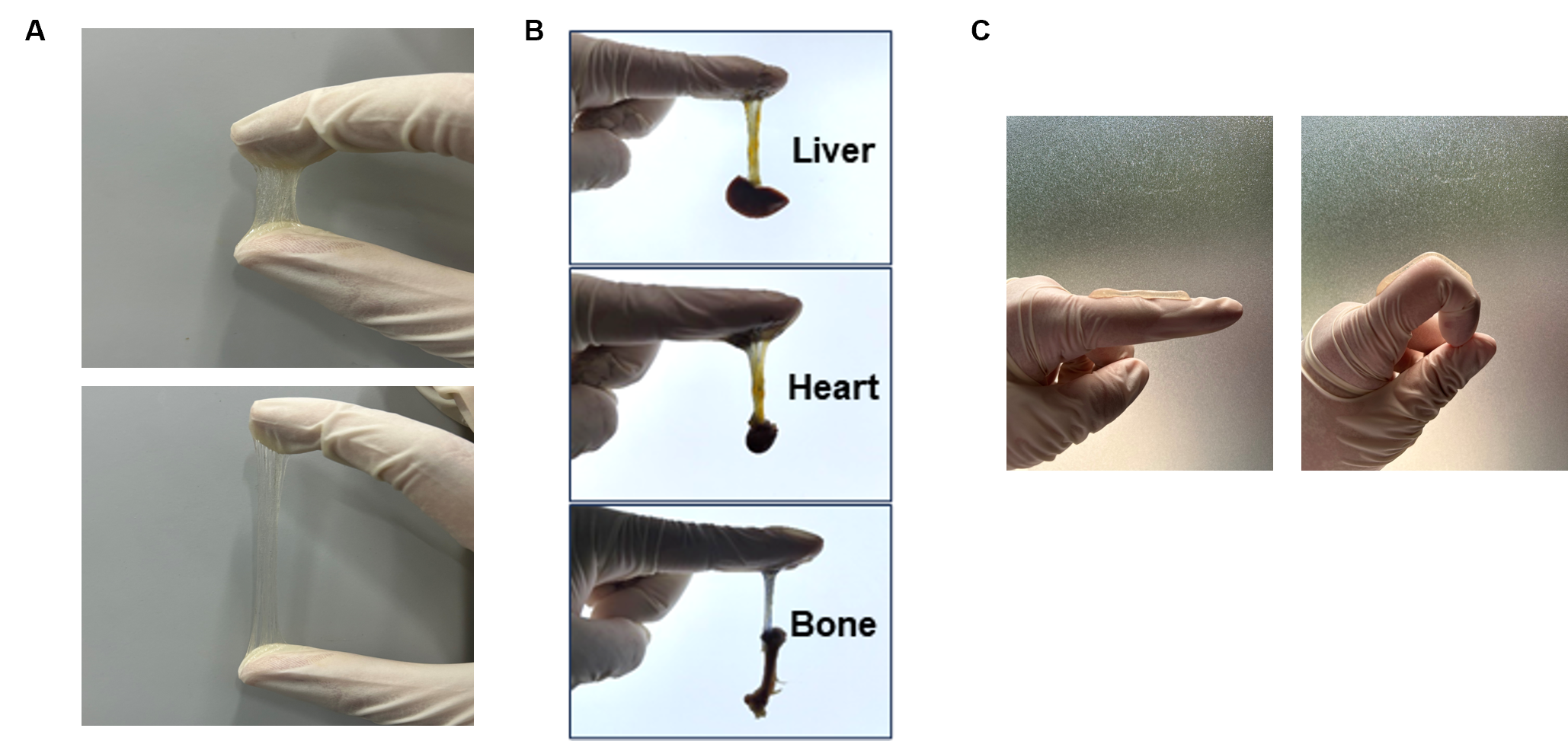


**Figure S4.** **A**) Tackiness of YDC-Gel-Zn bioglue. **B**) Tissue adhesion performance of YDC-Gel-Zn on liver, cardiac, and bone tissues. **C**) Stretchability of YDC-Gel-Zn bioglue.


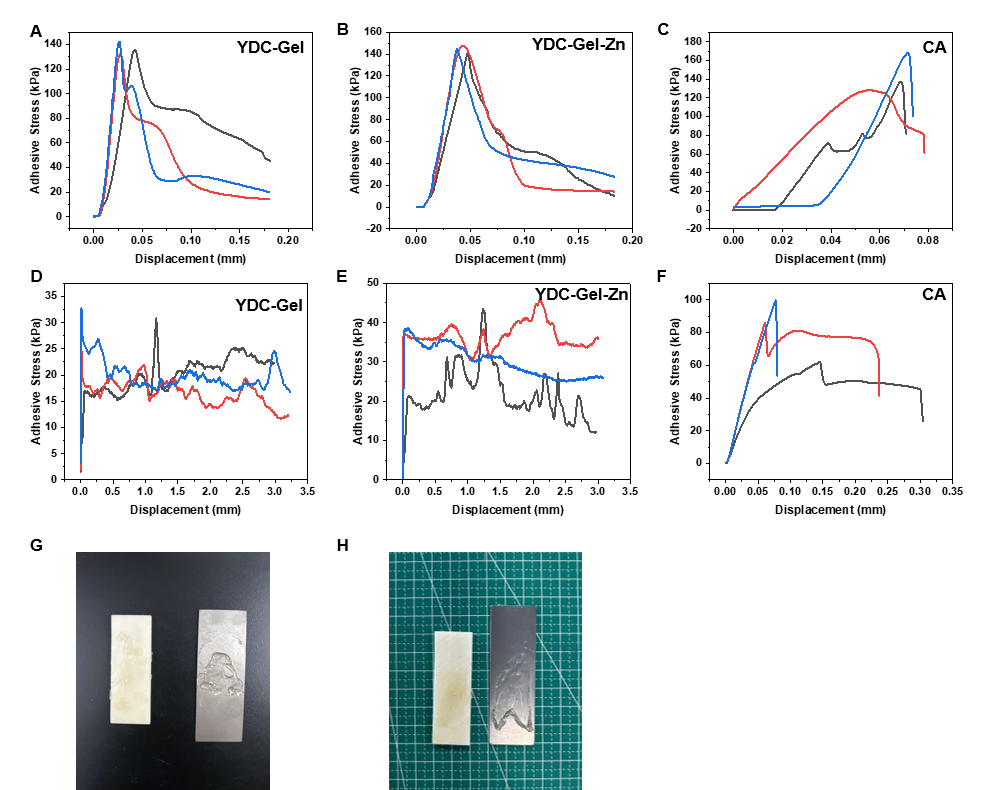


**Figure S5.** **A-C**) Tensile testing of bioglue at the bone plate-titanium sheet interface (n = 3). **D-F**) Shear testing of bioglue at the bone plate-titanium sheet interface (n = 3). **G**) General picture of the results of the tensile test. **H**) General picture of the results of the shear test.


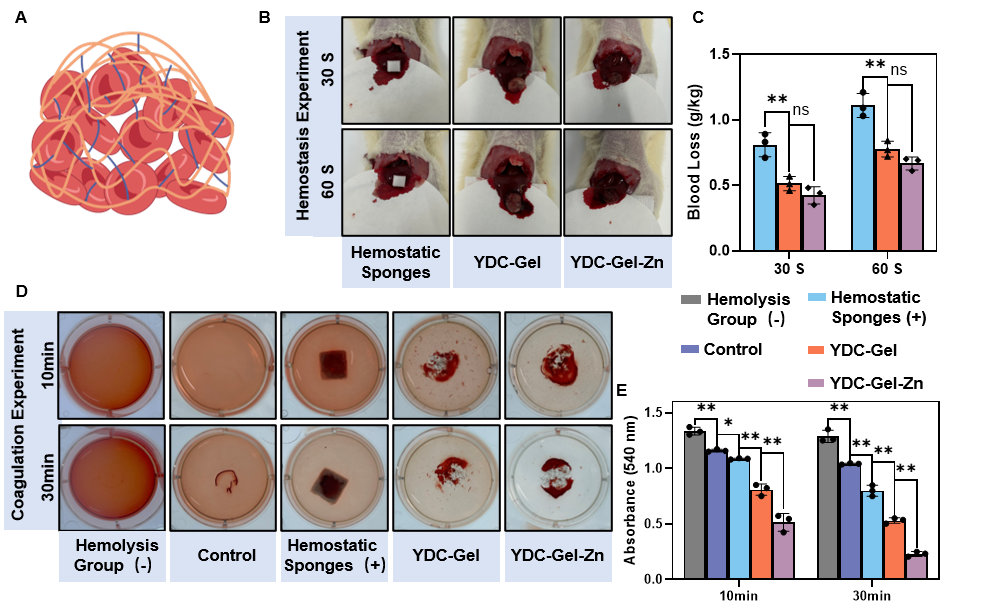


**Figure S6.** **A**) Schematic diagram of the hemostatic mechanism of YDC-Gel-Zn bioglue (created with BioRender.com). **B-C**) *In vivo* hemostatic efficacy comparison among commercial hemostatic sponges, YDC-Gel, and YDC-Gel-Zn (n = 3). **D-E**) *In vitro* blood coagulation assessment of commercial hemostatic sponges versus YDC-Gel and YDC-Gel-Zn (n = 3). The data are presented as the means ± SDs in the analysis figures, with *p < 0.05 and **p < 0.01 indicating statistical significance.


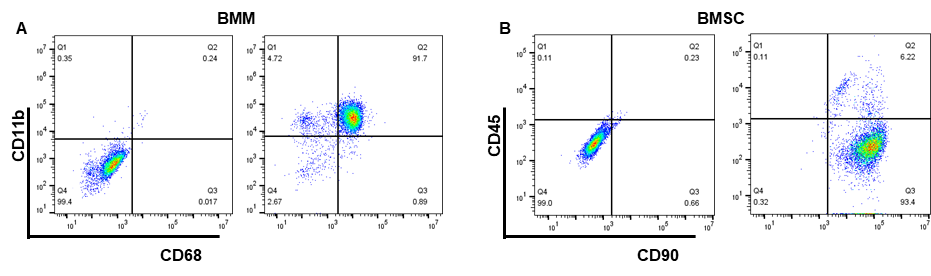


**Figure S7.A-B**) Flow cytometric analysis of primary BMM (CD68+, CD11b+) and BMSC (CD45-, CD90+) purity.


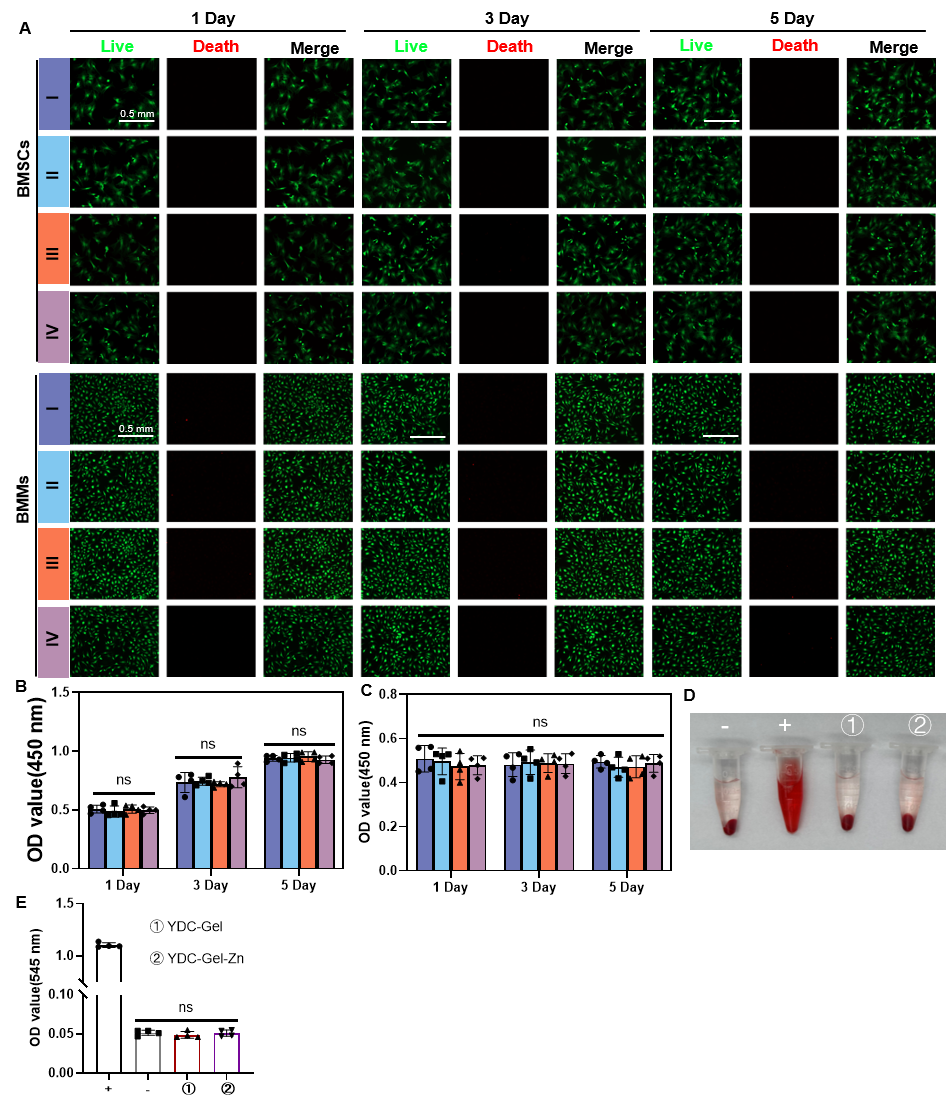


**Figure S8.** **A**) Representative diagrams of BMMs and BMSCs subjected to live/dead staining (n = 3) (I: Control; II: 0.33μm; III: 0.66μm; IV: 1.32μm). **B**) Effects of bioglue extract on the proliferation of BMSCs (n = 3). (**C**) Effects of bioglue extract on the proliferation of BMMs (n = 3). **D-E**) Hemolysis rates of red blood cells treated with different concentrations of PBS, ddH_2_O, YDC-Gel and YDC-Gel-Zn extracts (n = 3). The data are presented as the means ± SDs in the analysis figures, with *p < 0.05 and **p < 0.01 indicating statistical significance.


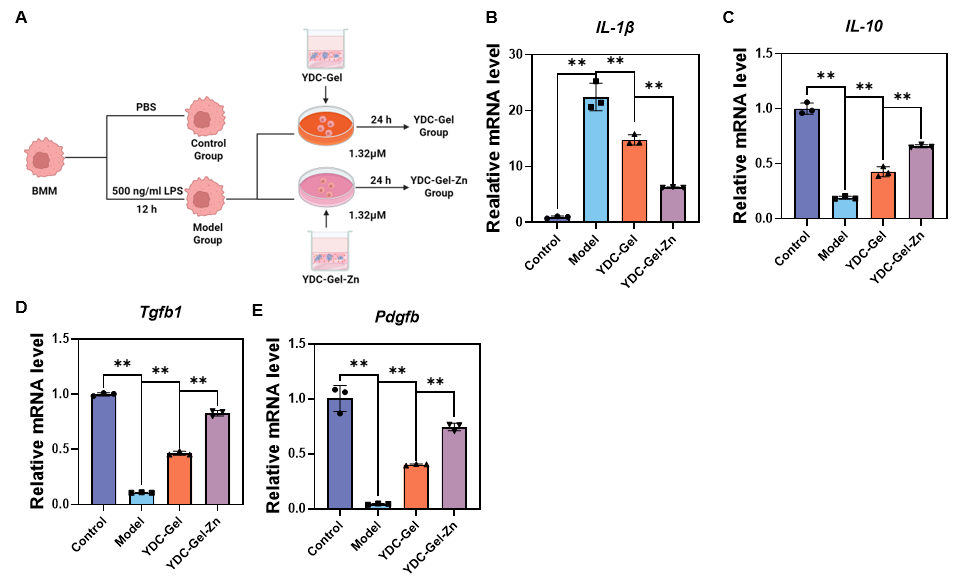


**Figure S9.** **A**) Schematic diagram of cell processing methods (created with BioRender.com). **B-E**) RT‒qPCR analysis of IL-1β, IL-10, Tgfb1 and Pdgfb expression levels in BMMs across different groups, along with quantitative results (n = 3). The data are presented as the means ± SDs in the analysis figures, with *p < 0.05 and **p < 0.01 indicating statistical significance.


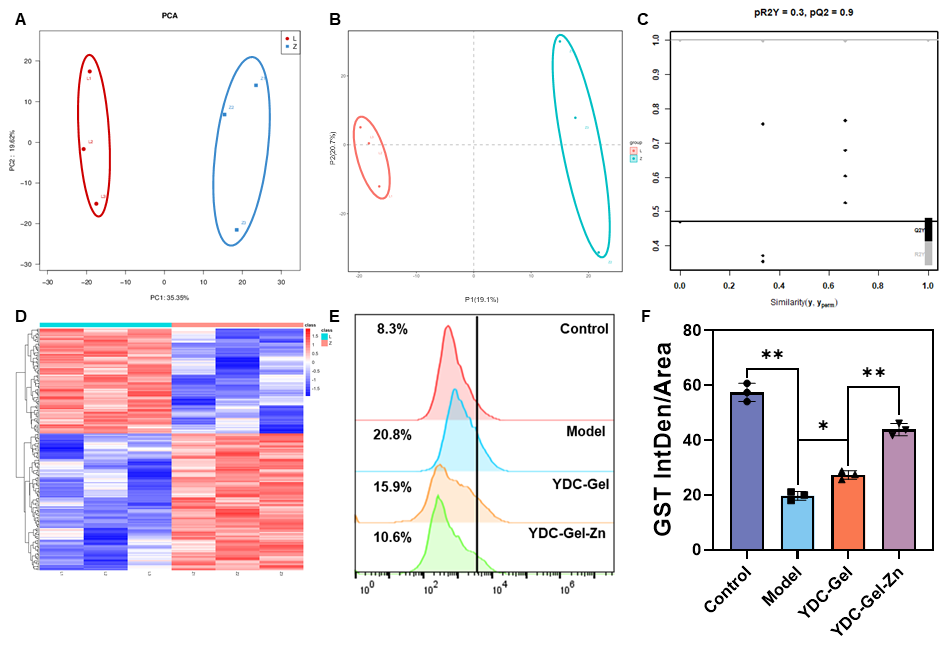


**Figure S10.** (**A**) PCA plot of high-throughput RNA sequencing samples. (**B-C**) PLS-DA results of metabolomics. (**D**) Heatmap of differentially expressed metabolites. (**E**) The fluorescence intensity of ROS in each group was analyzed by flow cytometry. (**F**) Quantitative analysis of immunofluorescence staining of GST (n = 3). The data are presented as the means ± SDs in the analysis figures, with *p < 0.05 and **p < 0.01 indicating statistical significance.

**
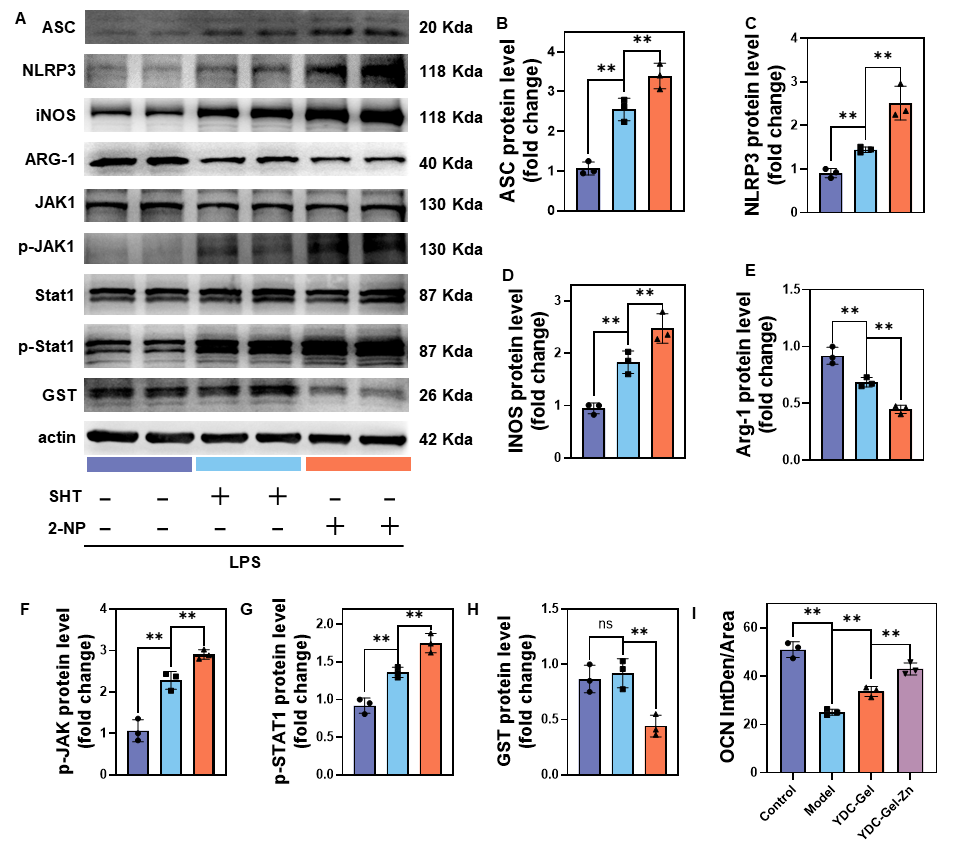
**

**Figure S11.** (**A-H**) Western blot analysis and quantification of proteins related to GST, the NOD-like receptor signaling pathway and the JAK1/STAT1 signaling pathway (n = 3). (**I**) Quantitative analysis of immunofluorescence staining of OCN (n = 3). The data are presented as the means ± SDs in the figures, with *p < 0.05 and **p < 0.01 indicating statistical significance.


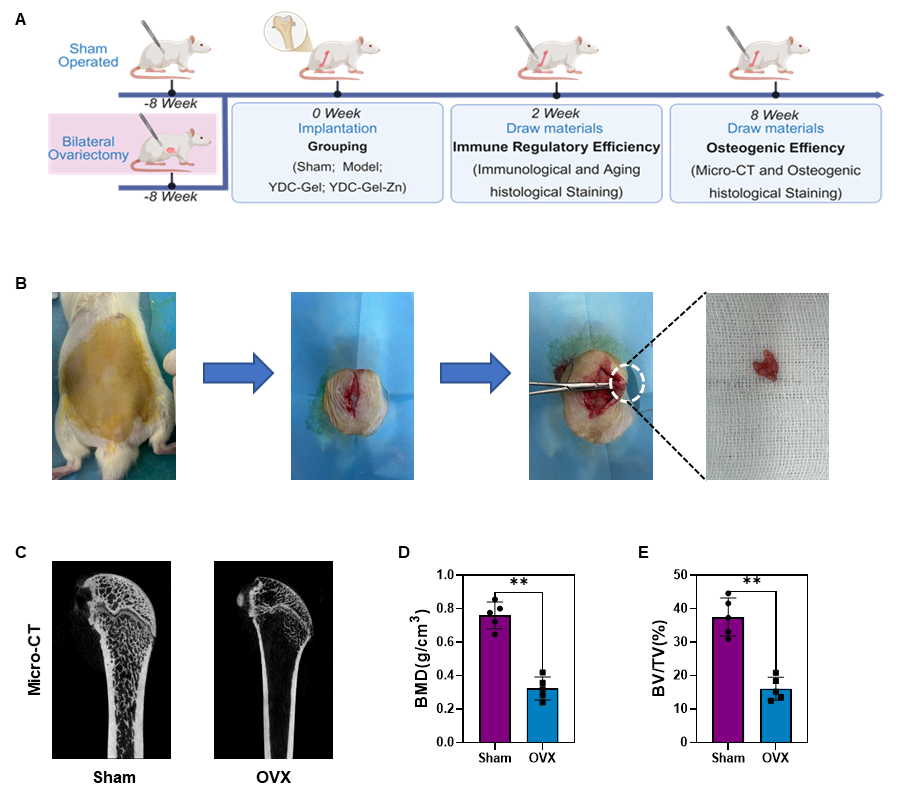


**Figure S12.** **A**) Schematic diagram of the animal experimental design (created with BioRender.com). **B**) Schematic diagram of rat ovariectomy surgery. **C**) Representative 3D reconstructed images of Sham group and OVX group. **D-E**) Quantitative parameters of trabecular bone in Sham group and OVX group (n = 5). The data are presented as the means ± SDs in the analysis figures, with *p < 0.05 and **p < 0.01 indicating statistical significance.

**
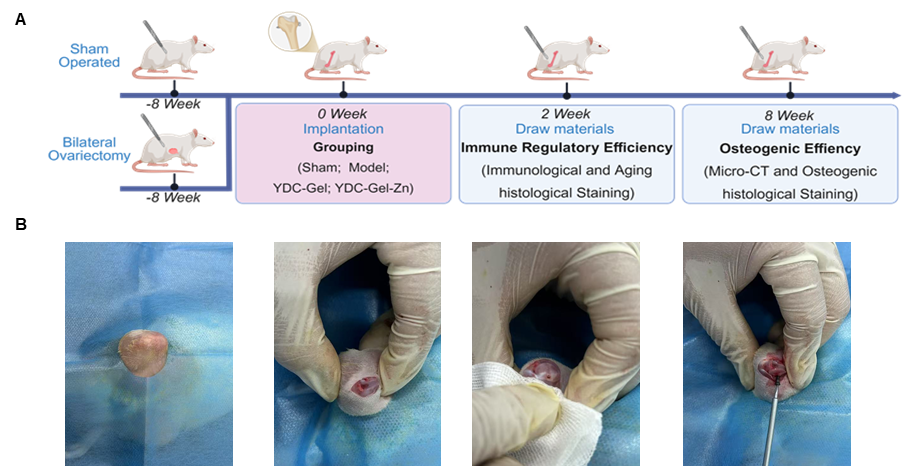
**

**Figure S13.** **A**) Schematic diagram of the animal experimental design (created with BioRender.com). **B**) Schematic diagram of screw implantation in the distal femur of rats.


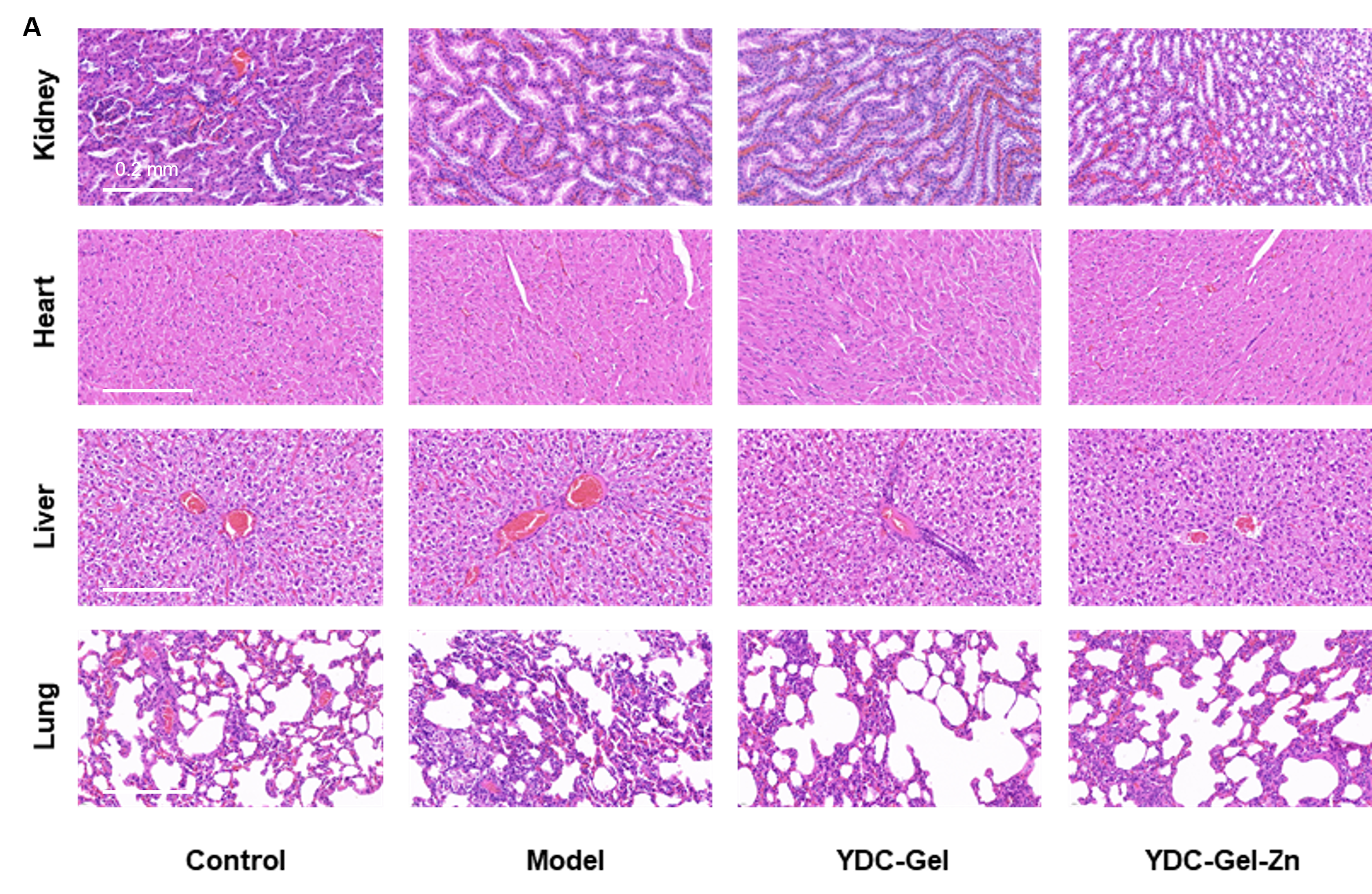


**Figure S14.** **A**) Representative HE-stained images of the kidney, heart, liver and lung in each group.


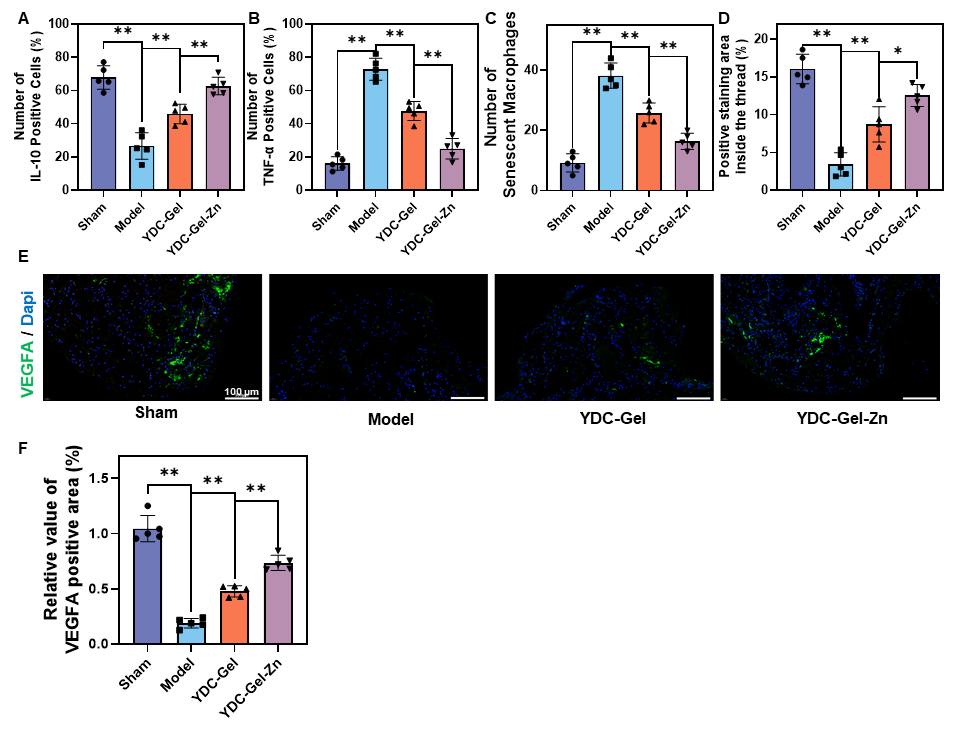


**Figure S15.** **A-B**) Proportion of IL-10^+^ and TNF-α^+^ cells in tissue fluorescence-stained sections (n = 5). **C**) Senescence-associated immunofluorescence quantitative analysis of the screw‒bone interface tissue (n = 5). **D**) Quantitative analysis of the proportion of toluidine blue-stained areas in the thread (n = 5). **E-F**) Representative images and quantitative analysis of Immunofluorescence staining of VEGFA at the screw-bone interface (n = 5).The data are presented as the means ± SDs in the analysis figures, with *p < 0.05 and **p < 0.01 indicating statistical significance.

**Supplementary Table S1.**

**Oligonucleotides used for Real-time PCR analysis**

| Gene (mus) | Forward Primers (5’-3’) | Reverse Primers (5’-3’) |
| --- | --- | --- |
| *Nos2*  *IL-1*β  *IL-10*  *Arg1*  *Actin*  *Csf2*  *Il6*  *Ccl2*  *Il1b*  *Cxcl2*  *Gadd45a*  *Cdkn1a*  *Trp53*  *Tgfb1*  *pdgfb* | GTTCTCAGCCCAACAATACAAGA  GAAATGCCACCTTTTGACAGTG  CTTACTGACTGGCATGAGGATCA  CTCCAAGCCAAAGTCCTTAGAG  CCTAGGCACCAGGGTGTGAT  GGCCTTGGAAGCATGTAGAGG  CTGCAAGAGACTTCCATCCAG  TAAAAACCTGGATCGGAACCAAA  GAAATGCCACCTTTTGACAGTG  CCAACCACCAGGCTACAGG  AGACCGAAAGGATGGACACG  CCTGGTGATGTCCGACCTG  CCCCTGTCATCTTTTGTCCCT  TCCATGACATGAACCGACCC  TGAAGACGAACCATCGGCTG | GTGGACGGGTCGATGTCAC  TGGATGCTCTCATCAGGACAG  GCAGCTCTAGGAGCATGTGG  GGAGCTGTCATTAGGGACATCA  AGCACAGGGTGCTCCTCA  GGAGAACTCGTTAGAGACGACTT  AGTGGTATAGACAGGTCTGTTGG  GCATTAGCTTCAGATTTACGGGT  TGGATGCTCTCATCAGGACAG  GCGTCACACTCAAGCTCTG  GTACACGCCGACCGTAATG  CCATGAGCGCATCGCAATC  AGCTGGCAGAATAGCTTATTGAG  GAAGTTGGCATGGTAGCCCT  GAACCAGCCAAGGCGTCTA |
